# A common promoter hypomethylation signature in invasive breast, liver and prostate cancer cell lines reveals novel targets involved in cancer invasiveness

**DOI:** 10.18632/oncotarget.5291

**Published:** 2015-09-22

**Authors:** David Cheishvili, Barbara Stefanska, Cao Yi, Chen Chen Li, Patricia Yu, Ani Arakelian, Imrana Tanvir, Haseeb Ahmed Khan, Shafaat Rabbani, Moshe Szyf

**Affiliations:** ^1^ Department of Pharmacology and Therapeutics, McGill University, Montreal, Quebec, Canada; ^2^ Departments of Medicine, Oncology, and Pharmacology, McGill University, Montreal, Quebec, Canada; ^3^ Department of Pathology, Fatima Memorial Hospital System, Lahore, Pakistan; ^4^ Department of Pharmacology and Therapeutics, Sackler Program for Epigenetics & Developmental Psychobiology, McGill University Medical School, Montreal, Quebec, Canada; ^5^ Department of Pharmacology and Therapeutics, Canadian Institute for Advanced Research, Montreal, Quebec, Canada; ^6^ Department of Nutrition Science, Purdue University, West Lafayette, Indiana, USA

**Keywords:** DNA methylation, epigenetics, invasiveness, drug targets, hypomethylation

## Abstract

Cancer invasion and metastasis is the most morbid aspect of cancer and is governed by different cellular mechanisms than those driving the deregulated growth of tumors. We addressed here the question of whether a common DNA methylation signature of invasion exists in cancer cells from different origins that differentiates invasive from non-invasive cells. We identified a common DNA methylation signature consisting of hyper- and hypomethylation and determined the overlap of differences in DNA methylation with differences in mRNA expression using expression array analyses. A pathway analysis reveals that the hypomethylation signature includes some of the major pathways that were previously implicated in cancer migration and invasion such as TGF beta and ERBB2 triggered pathways. The relevance of these hypomethylation events in human tumors was validated by identification of the signature in several publicly available databases of human tumor transcriptomes. We shortlisted novel invasion promoting candidates and tested the role of four genes in cellular invasiveness from the list *C11orf68, G0S2, SHISA2 and TMEM156* in invasiveness using siRNA depletion. Importantly these genes are upregulated in human cancer specimens as determined by immunostaining of human normal and cancer breast, liver and prostate tissue arrays. Since these genes are activated in cancer they constitute a group of targets for specific pharmacological inhibitors of cancer invasiveness.

**SUMMARY:**

Our study provides evidence that common DNA hypomethylation signature exists between cancer cells derived from different tissues, pointing to a common mechanism of cancer invasiveness in cancer cells from different origins that could serve as drug targets.

## INTRODUCTION

Invasion and growth regulation in cancer are distinctive processes and it stands to reason that they involve different sets of gene expression alterations driven by DNA methylation. A large body of studies implicated changes in DNA methylation in cancer [1–3]. The main focus has been on silencing of expression through hypermethylation of tumor suppressor genes and other genes that inhibit cancer progression. More recently several studies implicated activation of gene expression through hypomethylation of several pro-metastatic genes in breast [4], liver [5] and prostate cancer [6]. A recent analysis of cancer methylomes revealed that hypomethylation of CpG islands in breast cancer was associated with high metastatic risk and death [7]. DNA methylation profiles of cancer cells from different origins differ widely from each other. However, it stands to reason that fundamental mechanisms are shared amongst cancers from different origins and these should be captured in DNA methylation profiles even in cancer cell lines. In this study we focused on cancer cell invasiveness. We compared genome wide DNA methylation profiles of three invasive cancer cell lines derived from breast, liver and prostate cancers and their low invasive counterparts using Illumina 450K bead arrays and identified a common DNA methylation signature for invasive cell lines from different origins. We then determined whether such a signature is present in publicly available gene expression and DNA methylation data of metastatic tumor clinical samples.

We shortlisted new genes that were not previously described to be involved in cancer invasiveness and selected 4 genes for further analysis by examining their expression in cancer specimens and their role in cell invasiveness using *in vitro* invasion assays following shRNA knockdown. The results provide a proof of principle for this approach for identifying common targets for disrupting clinically relevant cancer phenotypes across cancers from disparate origins.

## RESULTS

### Common DNA methylation signatures in invasive prostate, breast and liver cancer cells

**Figure 1:**
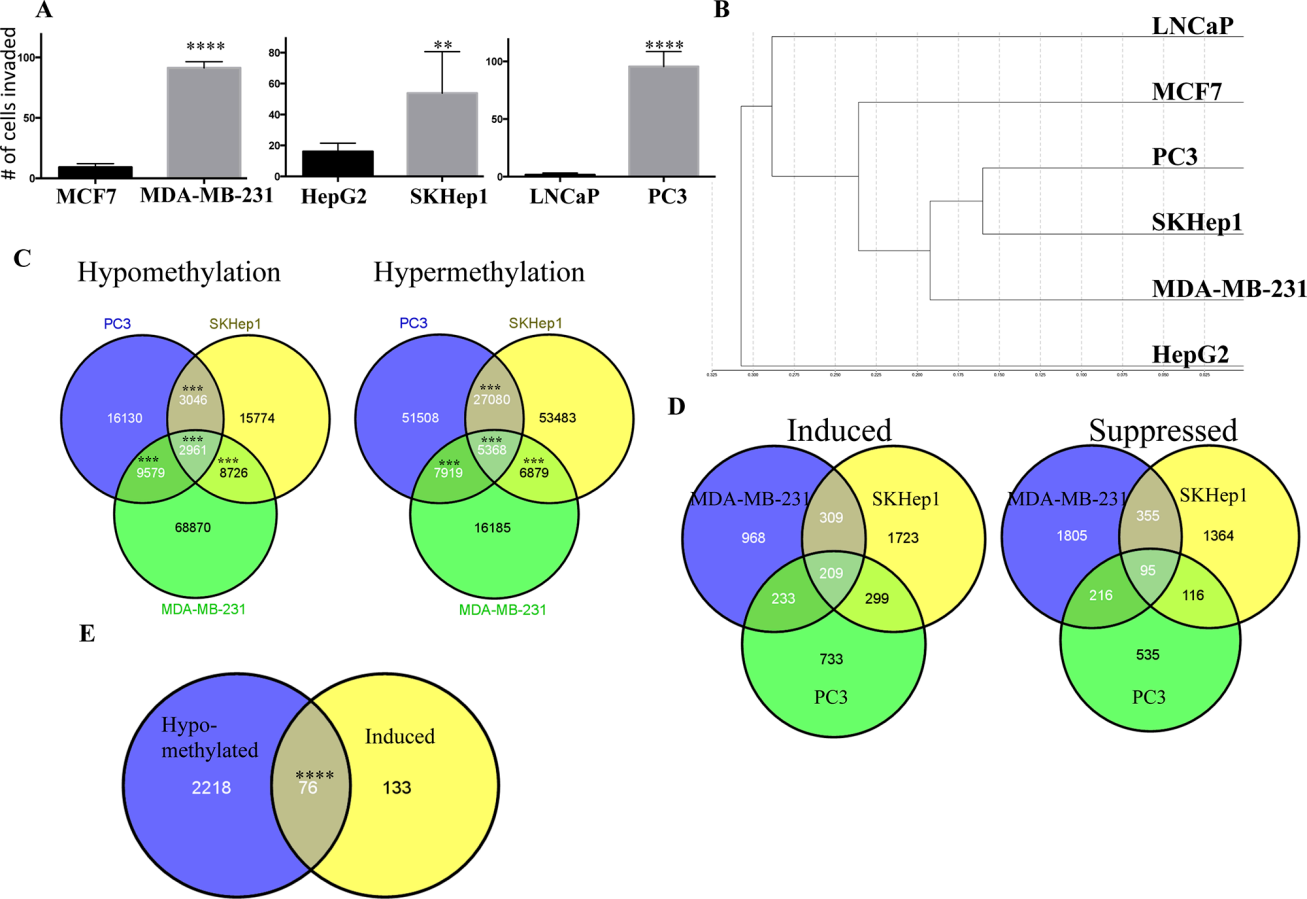
The DNA methylation landscape of metastatic cancer cell lines **A.** Invasiveness of cancer cell lines. The level of invasiveness of breast liver and prostate cancer cells was determined by a Boyden chamber invasion assay. Statistical significance determined by Student *t* test (***P < 0.0001; **P < 0.01) **B.** Hierarchical clustering of non-invasive (LNCaP, MCF7, HepG2) and invasive (PC3, SKHep1, MDA-MB-231) cell lines by their global methylation profiles of more than 450000 CpG sites **C.** Venn diagram showing overlapping methylation changes of CpG sites between three invasive cancer cell lines. Statistical significance of the overlap is indicated. A significant effect is represented as *** (*P* < 0.001). **D.** Venn diagram showing overlapping expression changes between three invasive cancer cell lines. **E.** Venn diagram showing overlapping hypomethylated and upregulated genes. Statistical significance of the overlap is indicated. A significant effect is represented as **** (*P* < 0.0001).

We compared the methylation profile of invasive cancer cell lines (breast MDA-MB-231, liver SKHep1, and prostate PC3) to their low-invasive counterparts (breast MCF7, liver HepG2, and prostate LNCaP) (Figure 1A). DNA from triplicate cultures (except LNCaP DNA, which was extracted from duplicate cultures) was subjected to whole genome DNA methylation analysis using Illumina 450K bead arrays as described in materials and methods. The Illumina 450K data has been submitted to GEO under accession number GSE71626.

We shortlisted the most robust differences in DNA methylation between the cell line pairs; differential DNA methylation of > 25% with *p*-value < 0.001. A hierarchical clustering analysis of the DNA methylation profiles of all 6 cancer cell lines grouped the invasive cancer cell lines MDA-MB-231, SKHep1 and PC3 in a common branch suggesting that the invasive cell lines are more similar to other invasive cell lines derived from a different tissue than to their own cell-type non-invasive cancer cell lines (Figure 1B). This is remarkable since the invasive cells are derived from different cancer origins and exhibit many different phenotypes. These data suggest a common DNA methylation profile of invasiveness across different cell types.

**Figure 2:**
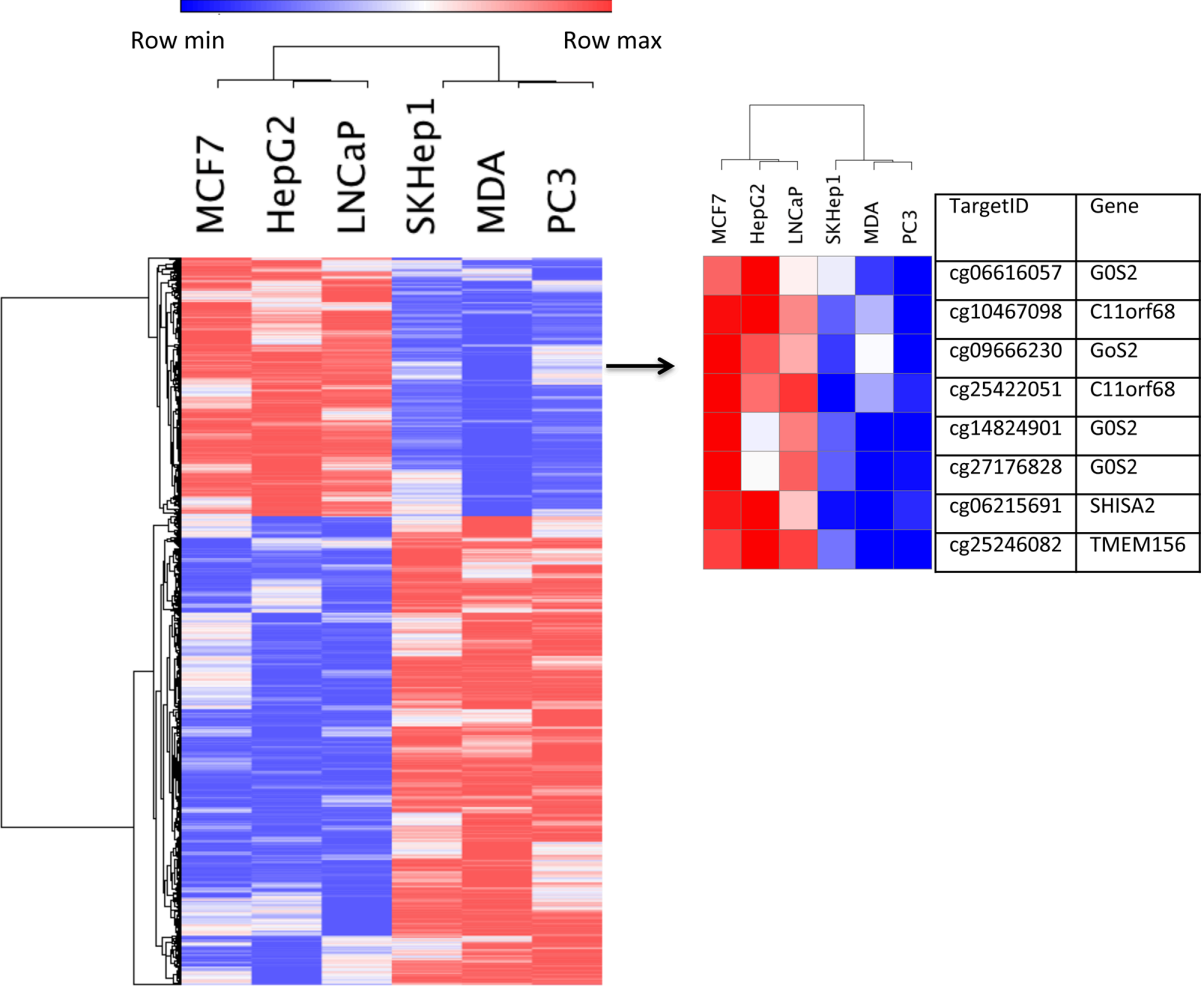
Gene methylation heatmap with a standard hierarchical clustering for 5368 differentially methylated in non-invasive (LNCaP, MCF7, HepG2) and invasive (PC3, SKHep1, MDA-MB-231) cancer cell lines (left) Right heat map represent selected out of left heat map target genes: *C11orf68, G0S2, SHISA2* and *TMEM156* and associated with them CpGs methylation heatmap (right).

**Figure 3:**
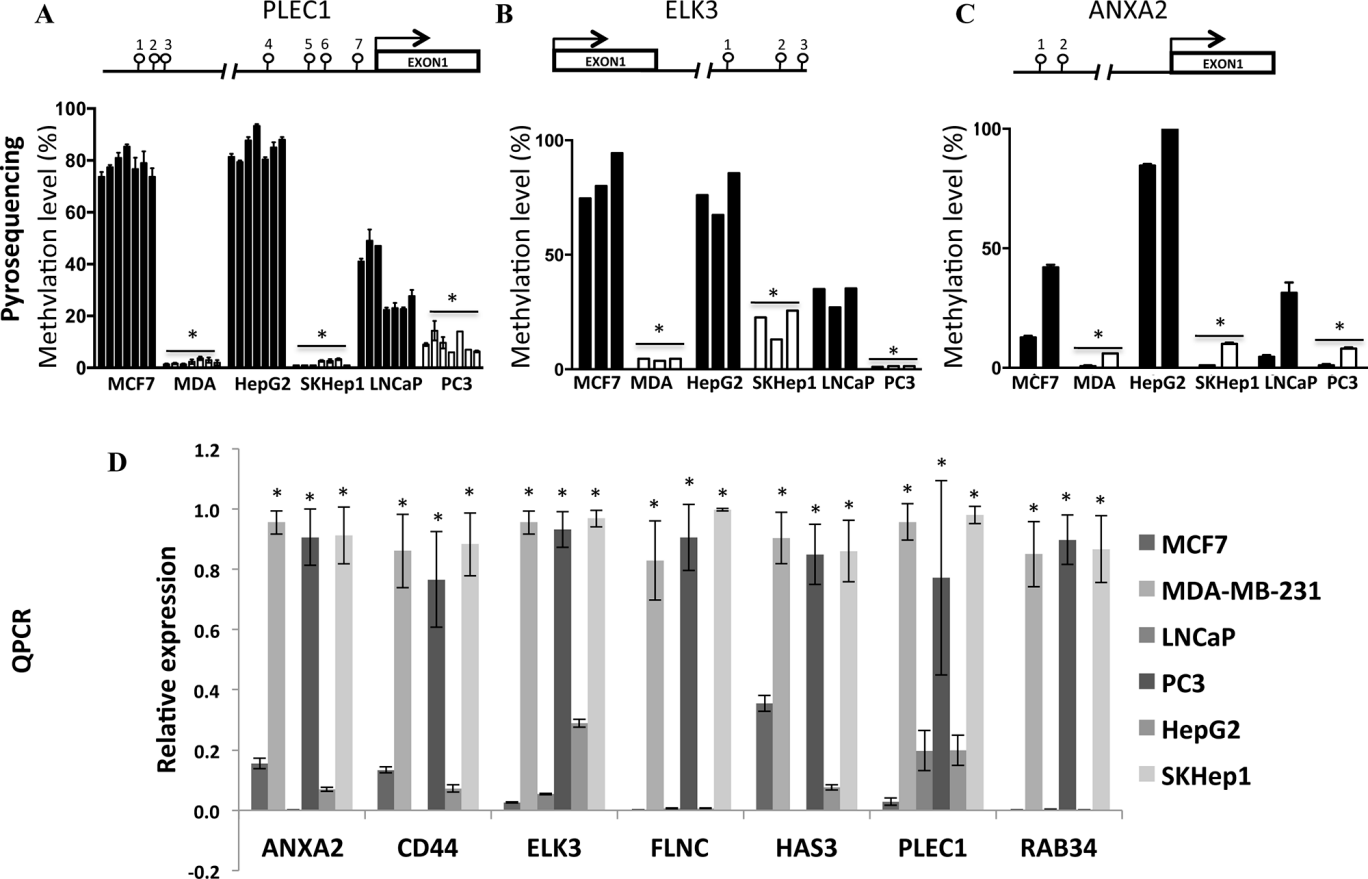
Validation of DNA methylation in PLEC1 A. ELK3 B. and ANXA2 C. and mRNA D. expression differences between invasive and low invasive cancer cell lines Pyrosequencing of A.) ANXA2 (NM_004039, Annexin A2), B.) ELK3 (NM_005230, ETS domain-containing protein) and C.) PLEC1 (NM_201380, Plectin) 5′UTR regions was performed on DNA extracted from invasive (MDA-MB-231, SKHep1, PC3) and non-invasive (MCF7, HepG2, LNCaP) cell lines. A schematic is shown for each 5′UTR with positions of CGs (lollipop) that were pyro sequenced indicated. Each bar represents the data for the individual CGs by the order presented in the scheme for each gene. Standard error is calculated from three experiments. Statistical significance was determined by Student *t* test. D.) Expression of ANXA2, CD44, ELK3, FLNC, HAS3, PLEC1 and RAB34 target genes in three non-invasive and three invasive cancer cell lines was determined by qPCR (see Materials and Methods). Experiment was performed at least three times in all three cell lines. Expression level of target genes was normalized to GAPDH rRNA values. Due to differences in the expression levels between the cell lines, the maximal expression level between replicates in invasive cells was matched to 1. The calculated ratios of invasive to non-invasive in all three type of cell lines were highly reproducible (see error bars).

We then computed the overlap between the differential DNA methylation signatures of the invasive versus non-invasive breast, liver and prostate cancer cell lines (Figure 1C). This analysis identifies a statistically significant overlap between the invasion specific DNA methylation signatures of the three cell types (Figure 1C). The analysis revealed 5368 CpG sites in 2075 genes that were significantly hypermethylated (*p* < 0.001) and 2961 CpGs in 1356 genes that were significantly hypomethylated (*p* < 0.001) in the three invasive cell lines relative to their cell-type non-invasive counterparts. Heatmap and hierarchical clustering of 5368 differentially methylated CpGs between invasive cells and their non-invasive counterparts, group invasive and non-invasive cancer cell lines in separate groups (Figure 2). Supplementary Table S2 provide comprehensive list of hypomethylated and hypermethylated sites. While most of the differentially methylated CpG sites are found in the open sea, 1172 hypermethylated sites (overlap significance: *P* = 9 × 10^−198^) and 542 hypomethylated sites (overlap significance: *P* = 5.8 × 10^−159^) are positioned 5′ to the genes, and are candidates to play a regulatory role in invasiveness. It is possible however that the differentially methylated CpG sites found in the open sea play other regulatory roles that are yet to be described. Interestingly, almost half (47%, 1395 out of 2961 CpG sites) (enrichment significance; *P* = 3.7 × 10^−266^) of all hypomethylated CpG sites in invasive cancer cell lines and 39% (2139 out of 5368 CpG sites) of the hypermethylated CpG sites occur in enhancer regions (enrichment significance; *P* = 7.3 × 10^−279^) but only 21% of CpGs in the Illumina 450K bead arrays are found in enhancers. We validated by pyrosequencing 3 randomly selected genes (*ANXA2, ELK3, PLEC1*) from the list of genes that were identified to be hypomethylated in their 5′ or promoter regions in our genome wide arrays in the invasive cells (Figure 3A, 3B, 3C). These 3 hypomethylated genes that were selected exhibited also significantly increased expression in invasive cancer cell lines as compared with the non-invasive counterparts as determined by QPCR (Figure 3D).

### Functional significance of Hypomethylated and Hypermethylated CpG sites in invasive cancer cell lines

We analyzed using Ingenuity Pathway Analysis (IPA) which gene networks, functional categories and canonical pathways are commonly differentially methylated in invasive cells across different cell types using a cutoff of a differential DNA methylation of at least 25% (Supplementary Table S2). Surprisingly, hypermethylated genes are organized in functional pathways that are only remotely related to cancer metastasis (Table 1). Remarkably, however the common hypomethylated landscape reveals some of the nodal pathways and regulators known to be involved in cancer metastasis (Table 1). The second biological function category after cancer includes cell movement (*P* = 5.18 × 10^−13^) one of the most important properties of cancer metastasis. That category includes 8 genes (*P* = 5 × 10^−3^) that are involved in Epithelial mesenchymal transitions (EMT) (Supplementary Table S3A) including *VIM* (*VIMENTIN*) [8], *ABL2/ARG* involved in defining the balance between proliferation and invasion [9] in both cell culture and animal experiments, *PAPPA* [10] a protease required for invasiveness of several cancer cell lines, and *uPA* (urokinase), a well defined marker of invasive tumor cells that is regulated by methylation of its promoter region [11]. In general, the common hypomethylated genes in invasive cancer cell lines are known to be crucial for cell movement, proliferation, migration and invasion (Supplementary Table S4).

**Table 1:** IPA of differentially methylated genes in invasive compared with non-invasive cancer cell lines

Functional analysis of 1172 genes with hypermethylated promoters regions		
Biological processes	*p*-Value	# Genes
Cell-To-Cell Signaling and Interaction	6.7 × 10^−12^ −5.7 ×10^−3^	211
Cell Signaling	1.08 × 10^−7^ −4.92 × 10^−3^	103
Nucleic Acid Metabolism	2.04 × 10^−7^ −4.92 × 10^−3^	53
Small Molecule Biochemistry	2.04 × 10^−7^ −5.67 × 10^−3^	133
Cell Death and Survival	4.69 × 10^−7^ −4.92 × 10^−3^	204
**Functional analysis of 542 genes with hypomethylated promoters regions**		
Cellular Growth and Proliferation	5.4 × 10^−14^ −4.2 × 10^−3^	191
Cellular Movement	5.18 × 10^−13^ −4.0 × 10^−3^	128
Cell Death and Survival	8.78 × 10^−12^ −4.19 × 10^−3^	171
Cellular Development	1.87 × 10^−10^ −4.12 × 10^−3^	166
Cellular Assembly and Organization	7.22 × 10^−8^ −3.88 × 10^−3^	85

Functional analysis of 1172 genes that were significantly hypermethylated (*p* < 0.0001) and of 542 genes that were significantly hypomethylated (*p* < 0.0001) more than 25% in 5′UTR gene region in invasive compared with matched non-invasive cancer cell lines.

235 genes included in the signature of commonly hypomethylated genes in invasive cancer cells described here were not previously shown to our knowledge to be involved in cancer or cancer metastasis. However, the biochemical function of these genes might suggest possible involvement in cancer. For example 16 genes (Supplementary Table S3B) were shown to be involved in TGF beta pathway that is known to drive cancer metastasis [12]. These data support the hypothesis that common DNA methylation changes underlie the invasive phenotype across diverse cell types and that these common DNA methylation signatures capture genes that are critical for the invasive phenotype in many cancers.

To get further insight into the functional organization of the hypomethylation signature we examined putative upstream regulators of the commonly hypomethylated genes. The list includes downstream effectors of prime drivers of cancer, such as HRAS, and cancer metastasis, such as TGF beta. The involvement of TGF beta pathway in driving cancer metastasis, particularly breast cancer metastasis has been widely documented in the last two and a half decades [13, 14]. There is evidence that TGF beta stimulates EMT transition, the primary process converting non-invasive epithelial cells into invasive carcinoma [15, 16]. Interestingly, a careful examination of the list of hypomethylated genes that are targets of TGF beta reveals 5 genes (*PLAU, PLAUR, SMAD3, STAT3* and *VIM*) involved in EMT transition (reviewed in [17]).

Another notable upstream regulator is the receptor tyrosine kinase *ERBB2* (*HER2*) [18]. Thousands of publications have implicated this protein in breast cancer metastasis. *HER2* overexpression occurring in aggressive metastatic breast cancer, is the principal somatic amplification/overexpression [19] known to be a marker of disease outcome and therapeutic response in breast cancer [20, 21] and a target of approved drugs for the treatment of metastatic breast cancer [22–24]. Interestingly *ERBB2* itself is not differentially methylated.

### Validation of the relevance of the hypomethylation signature to clinical samples

We compared our differentially methylated genes profile to publicly available Illumina 450K methylation results from 22 pure DCIS (ductal carcinoma *in situ*), 31 mixed DCIS-IBC (ductal carcinoma *in situ*-invasive ductal carcinoma) and 186 pure IBC (invasive breast carcinoma) [25]. First we found significant overlap (*p*-value = 0.03) of 26 genes (Table S5) when we compared our list of the common signature of differentially methylated genes in invasive cancer cell lines to 154 genes that are differentially methylated between DCIS (ductal carcinoma *in situ*) and IDC (invasive ductal carcinoma) (GSE60185) [25]. More significant (*p*-value = 9 × 10^−07^) results (114 common genes) were obtained (Table S5), when we compared the list of differentially methylated genes between invasive (MDA-MB-231) and non-invasive (MCF7) breast cancer cells and the154 differentially methylated genes between DCIS and IDC. We couldn't find data for differentially methylated genes between invasive and non-invasive cancers in liver and prostate tumors in the public domain.

Hypomethylation of 5′ UTR of genes is believed to be associated with activation of gene expression. We therefore examined the overlap between the list of hypomethylated genes in invasive cancer cell lines and genes upregulated in tumor gene expression databases. A comparison of the list of hypomethylated genes (*P* < 0.05, delta beta < − 0.25) in invasive PC3 prostate cancer cell line with upregulated genes in publicly available microarray expression data from 216 tissue samples collected from 51 patients with prostate cancer (accession in ArrayExpress: E-GEOD-3325) [26] reveals an overlap of 1440 genes (*p*-value = 1.21 × 10^−292^). These genes are hypomethylated in invasive PC3 prostate cancer cell lines and are overexpressed in invasive prostate cancer (Supplementary Table S6). Similarly, 199 genes that are hypomethylated in MDA-MB-231 cells vs. MCF-7 cells are included in a list of 349 upregulated genes in Invasive Ductal Carcinoma in comparison to Ductal Carcinoma *in Situ* (GSE3893) (*P* < 1.85 × 10^−36^) (Supplementary Table S6) [27]. 371 genes that are hypomethylated in invasive SKHep1 liver carcinoma cells vs. HepG2 cells overlap with a recently published DNA demethylation landscape of highly aggressive HCC from patients in China [5] (*p*-value = 1.93 × 10^−09^) (Supplementary Table S6). DNA methylation data comparing invasive and non-invasive liver and prostate cancer tumor samples was unavailable in public released data.

In summary, these analyses suggest that the approach that we have taken in this study identifies important genes for invasiveness in tumor samples and is not limited to cell culture conditions.

### Discovery of new candidate genes involved in cell invasiveness

We first delineated differentially methylated genes that were also differentially expressed between the invasive and non-invasive cancer cell lines by subjecting RNA extracted from the MCF7, MDA-MB-231, HepG2, SKHep1 cells to expression analyses using the Illunima HT12 bead array platform. The Illumina HT12 expression beadchip arrays data has been submitted to GEO under accession number GSE71625. For prostate cancer cell lines (PC3 and LNCaP), we used publicly available data (GSE36531) [28]. We found 95 significantly (*P*-value 0.05, larger than twofold difference) downregulated and 209 upregulated genes in all three invasive cancer cell lines compared to non invasive cancer cell lines (Figure 1D). We determined the overlap between genes that were hypomethylated in 5′UTR regions and upregulated genes in invasive compared to non-invasive cancer cell lines. We found significant (*p*-value = 3.65 × 10^−45^) overlap of 76 RefSeq genes including isoforms covering 69 genes between the hypomethylated and overexpressed genes in invasive cancer cell lines (Figure 1E) (For the whole list see Supplementary Table S7). 31 out of these 76 genes are known to be involved in metastasis, while 46 to our knowledge were not reported previously to be involved in metastasis formation.

To provide a proof of principle that the list of genes that are commonly hypomethylated and induced in 3 invasive cancer cell lines could serve as a source for discovery of new genes involved in invasiveness, we picked four genes, *C11orf68, G0S2, SHISA2* and *TMEM156*.

**Figure 4:**
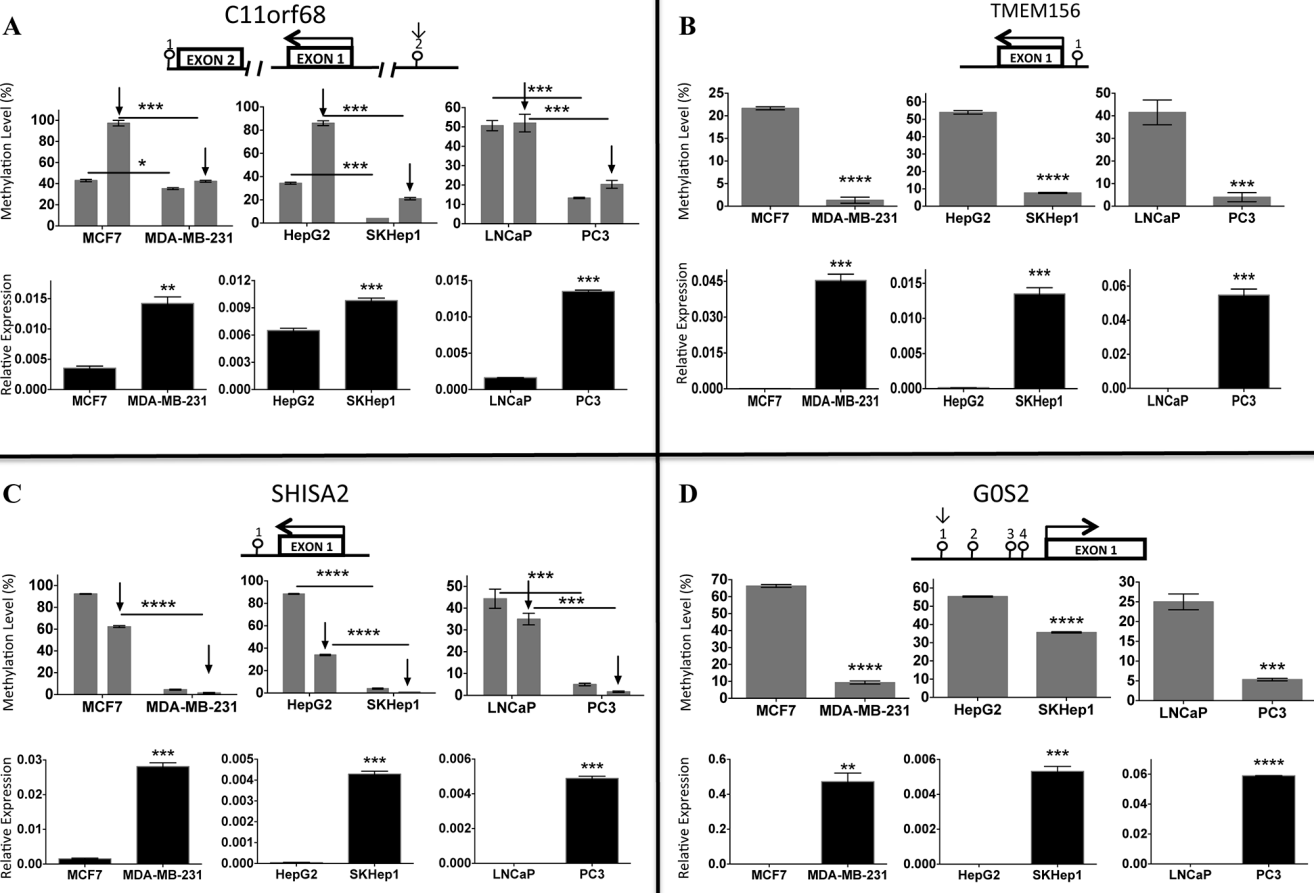
Expression and Pyrosequencing of *C11orf68 A. TMEM156 B. SHISA2 C. and G0S2 D. and* target genes in invasive and non-invasive cell lines was determined by QPCR and Pyrosequencing respectively (see Materials and Methods) Experiments were performed in triplicate in invasive (MDA-MB-231, SKHep1 and PC3) and their non-invasive (MCF7, HepG2 and LNCaP) counterpart cell lines. Expression levels of target genes were normalized to *GAPDH* rRNA values. A schematic diagram is shown for each of the four genes: *C11orf68 (A), TMEM156* (B), *SHISA2 (C)* and *G0S2 (D)*. Positions of CpGs (lollipop) that were sequenced are indicated. Standard error is calculated from three experiments. The number above lollipop is corresponding to the ID obtained from Illumina 450K for each studied CpG as follows: C11orf68 (1- cg25422051, 2- cg10467098) (A); TMEM156 (cg25246082) (B); SHISA2 (cg06215691) (C), G0S2 (1- cg06616057, 2-cg09666230, 3-cg14824901, 4-cg27176828) (D) The arrows indicate the position of CGs from the Illumina450K array that were validated. The Y axis indicates the percentage methylated cytosines according to each CpG shown on X axis. A significant effect is represented as ****, *P* < 0.0001; ***, *P* < 0.001; **, *P* < 0.01; *, *P* < 0.05.

These genes were chosen for the following reasons. First, to our knowledge they were not previously reported to be involved in cell invasiveness. Second, differences in expression of these genes were robust (*P* value < 0.001). Third, the biological function of two out of the four genes (*C11orf68*-Basophilic Leukemia-Expressed Protein and *TMEM156)* was unknown. *G0S2* is G0/G1switch 2 protein, known to be involved in cell proliferation and apoptosis [29], differentiation [30], inflammation [31], and lipid metabolism [32] in various cellular settings. *SHISA2* plays an essential role in the maturation of presomitic mesoderm cells by individual attenuation of both FGF and WNT signaling [33]. Steady state mRNA levels of *C11orf68, G0S2, SHISA2* and *TMEM156* were significantly upregulated in invasive MDA-MB-231, SKHep1 and PC3 cell lines compared to their non-invasive counterparts as validated by QPCR (Figure 4) and their promoters were hypomethylated as validated by pyrosequencing (Figure 4). (Please note, that due to the lack of TMEM156 antibody suitable for western blot analysis, we were not able to test this gene).

### Expression of new candidate genes in cancer and normal tissues

We determined whether these genes were overexpressed in clinical cancer biopsies in comparison with cancer adjacent normal and/or normal tissues by immuno-histochemistry using specific antibodies targeted against these proteins (Figure 4) (for details, please see Material and Methods).

The vast majority of our clinical samples were from invasive cancers and we were only able to determine the expression of the proteins in invasive cancer compared to normal and normal adjacent tissues. (Please note, that due to the lack of G0S2 antibody available for immunostaining, we were not able to test this gene).

**Figure 5:**
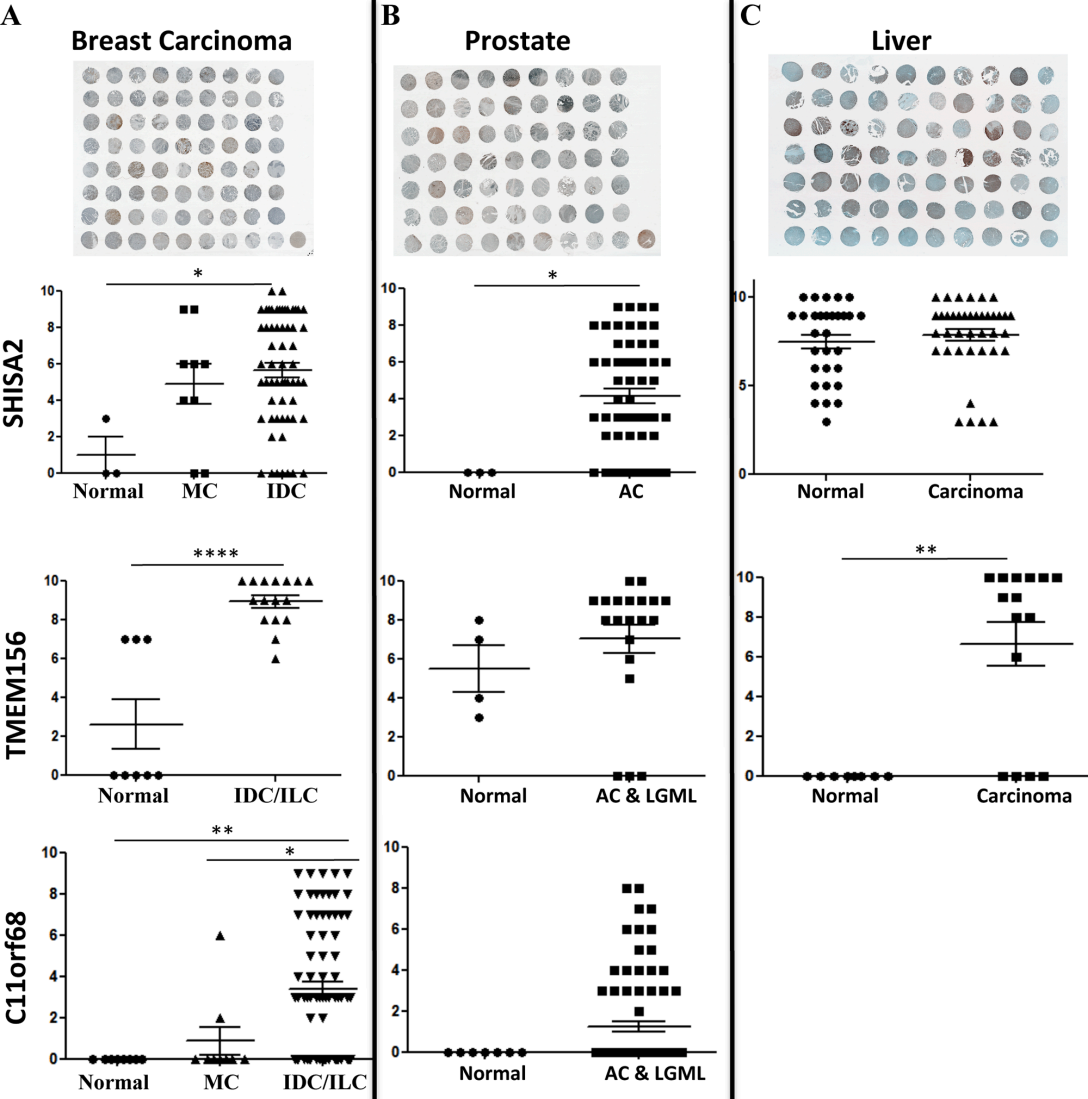
Immunohistochemistry analysis of *C11orf68, G0S2, SHISA2* and *TMEM156* expression in breast A. prostate cancer B. and liver C Tissue specimens obtained from US Biomax Inc. (Rockville, MD) from normal (normal tissues from healthy individuals **(N)** and/or cancer adjacent normal tissues-NAT); breast medullary (MC), invasive ductal (IDC) and lobular (ILC) carcinomas; liver hepatocellular carcinoma (HC); prostate adenocarcinoma (AC), low grade malignant leiomyosarcoma (LGML) were stained with specific antibodies (see Materials and Methods for details). The staining intensity represented on the Y-axis represents the total score, calculated as described in Material and Methods. Example images from Biomax samples collected from each studied tissue: breast, liver and prostate are depicted above the charts. A significant effect is represented as ****, *P* < 0.0001; ***, *P* < 0.001; **, *P* < 0.01; *, *P* < 0.05.

***C11orf68***. Higher staining was detected in invasive lobular (*n* = 4) (*p* < 0.05) and ductal carcinoma (*n* = 75) compared to normal and cancer adjacent normal tissues (NAT) (*n* = 8) (*p* < 0.01) (Figure 5A) (Supplementary Table S8). Interestingly, the expression of *C11orf68* was higher in invasive ductal and lobular carcinomas compared to medullary carcinoma (*n* = 9) (*p* < 0.05), which is known to be the only carcinoma associated with *BRCA1* mutation. We did not observe a significant difference between medullary carcinoma and normal and NAT tissues (Figure 5A). We did not detect any expression of C11orf68 in normal prostate tissues (*n* = 7) and low grade malignant leiomyosarcoma tissues (*n* = 9), however 22 out of 80 prostate adenocarcinoma tissues were positively stained (Figure 5B) (Supplementary Table S8).

### SHISA2

Higher staining was detected in invasive breast ductal carcinoma (*n* = 59, one sample of invasive ductal carcinoma (fibropatty tissue) was excluded, since no tumor was observed) as compared to normal adjacent tissue. In medullary carcinoma *SHISA2* high expression was observed in 7 out of 9 patients tissues as compared to NAT (*P* = 0.084) (Figure 5A) (Table S8). SHISA2 was also expressed at higher level in hepatocellular carcinoma (*n* = 39) as compared to NAT (*n* = 13) (*P* < 0.001). Surprisingly, normal tissue (*n*-17) gave high expression of *SHISA2* compared to NAT (*p* < 0.001) and differences with hepatocellular carcinoma were not significant (Supplementary Table S8) (Figure 5C).

*SHISA2* was significantly elevated in prostate adenocarcinoma (*n* = 56) samples as compared to normal prostate tissue (Biomax, PR633) (*P* < 0.05). 44 out of 56 adenocarcinoma tissues were positively stained with SHISA2 antibody (Figure 5B) (Supplementary Table S8).

### TMEM156

There was strong positive staining in invasive ductal and lobular breast carcinoma (*n* = 14) as compared to normal breast tissue (*n* = 4) (*P* < 0.00001) and NAT (*n* = 4) (*P* < 0.01) (Figure 5A) (Supplementary Table S8). While no staining was detected in normal liver tissue (*n* = 8), liver cancer samples (*n* = 16) showed significant induction of TMEM156 (*P* < 0.001) (Supplementary Table S8). In prostate adenocarcinoma the average staining intensity in normal tissues was lower compared to adenocarcinoma stain (5.5 vs. 7) however this difference was not significant (*P* = 0.38) (Figure 5B) (Table S8) (see summary of expression data for all clinical samples and information on the tissue arrays used in Supplementary Table S8).

These data suggest that the selected three proteins are overexpressed in some but not all invasive tumor types that were analyzed.

### Functional testing of the role of hypomethylated gene in invasiveness of cancer cells

**Figure 6:**
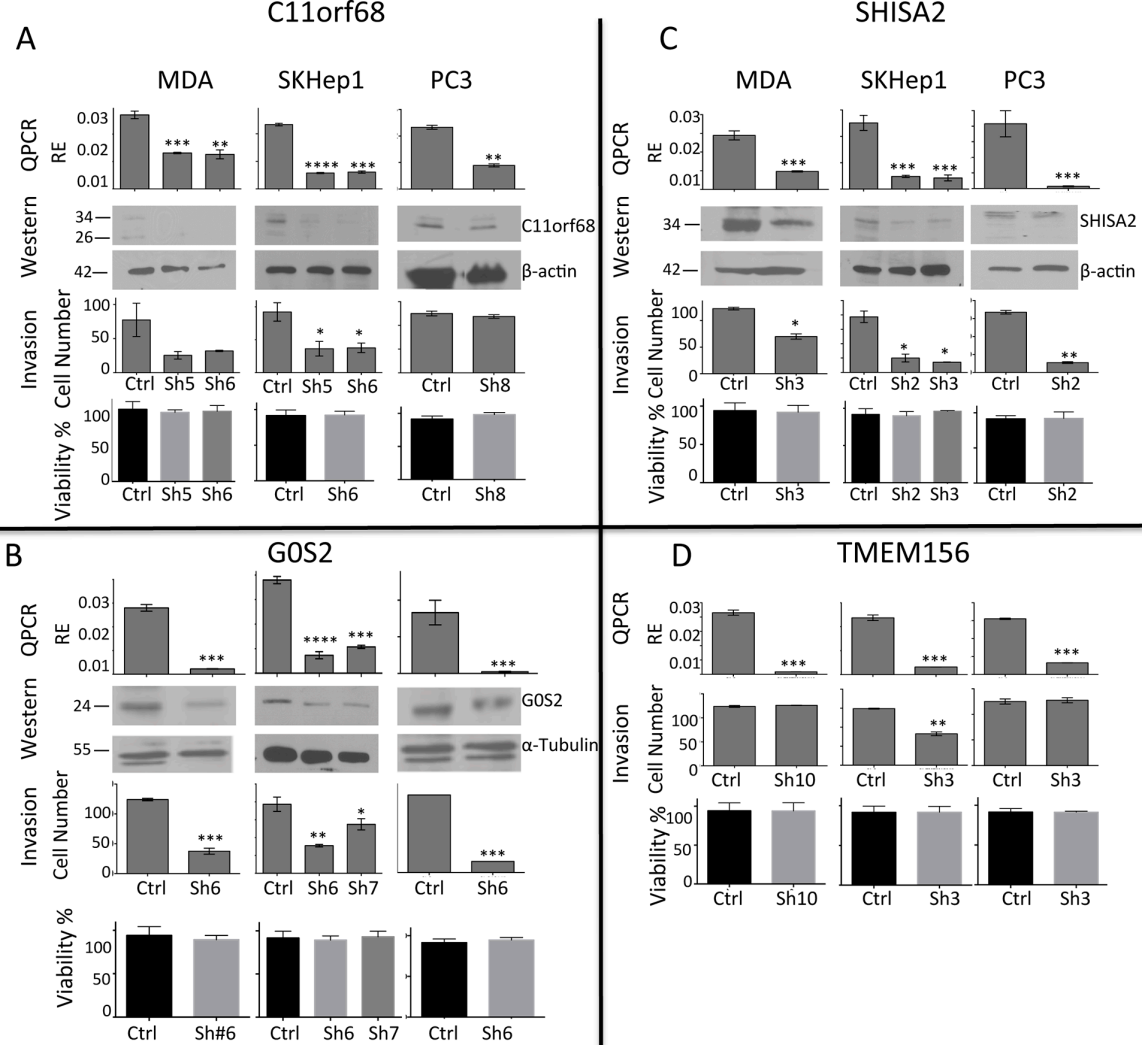
*C11orf68, G0S2, SHISA2 and TMEM156* depletion decreases invasive capacities *in vitro* without affecting cell viability Invasive breast (MDA-MB-231), liver (SKHep1) and prostate (PC3) cancer cell lines were stably transfected with different shRNAs targeting *C11orf68* **A.** *G0S2* **B.** *SHISA2* **C.** and *TMEM156* **D.** Expression (designated as “QPCR” and “Western” in A, B, C, D) of the depleted genes, quantified by QPCR and western blot after infection of MDA-MB-231, SKHep1 and PC3 with scrambled shRNA (ShSCR) and shRNAs as listed above directed to each of the tested genes. Quantitative analysis of western blots was performed using Image J software. C11orf68, G0S2, SHISA2 expression levels over b-actin levels are presented (TMEM156 antibody for western blot is not available). Invasion assays (designated as “Invasion” in A, B, C, D) were performed on transfected MDA-MB-231, SKHep1 and PC3 in Boyden Matrigel Invasion chambers for 48 h for PC3 and 24 hours for MDA-MB-231 and SKHep1 as described in ‘Materials and Methods’. Data represent an average ± SD of the mean of an experiment performed in triplicate experiments. Cell invasion was measured by Boyden chamber invasion assay. All results represent mean ± S.D. of three determinations in either two or three independent experiments; ***P < 0.001, **P < 0.01, *P < 0.05. Cell viability of the treated cells that were plated in 24 wells that did not contain a Matrigel-coated membrane.

To test whether *C11orf68, G0S2, SHISA2* and *TMEM156* are required for the invasive phenotype of breast MDA-MB-231, liver SKHep1and prostate PC3 cancer cell lines, we used stable shRNA-mediated depletion of these genes with lentiviral particles expressing shRNA directed against these 4 genes (3 different pools of stable infected cells were tested per shRNA construct). The shRNA depleted cell lines showed reduced expression of the genes at both mRNA and protein levels as confirmed by QPCR and western blot (Figure 6A, 6B, 6C, 6D).

Trypan blue-excluding cells were quantified using a hemocytometer, and an equal amount of viable cells were plated per group in Boyden chambers to measure *in vitro* invasiveness (see Material and Methods for details).

Depletion of *C11orf*68 (Figure 6A) resulted in inhibition of invasiveness in breast MDA-MB-231 and liver SKHep1 cell lines but not in PC3 prostate cancer cells. *G0S2* and *SHISA2* (Figure 6B, 6C) depletion caused significant reduction in invasiveness in all three invasive cell lines and knockdown of *TMEM156* had an effect on invasiveness only in SKHep1 cells (Figure 6D). In summary, knockdown of all 4 genes results in inhibition of invasiveness in at least one of the three invasive cell lines.

To exclude an impact of RNAi on cell viability that may confound the invasive assay results, we plated the treated cells concurrently on similar wells but in absence of the Matrigel-coated membrane and performed cell Trypan blue cell viability assay. We did not observe any significant effect on cell viability by knockdown of indicated genes supporting the hypothesis that the selected genes are involved in cancer cell invasiveness but not cancer cell growth or apoptosis illustrating divergence of mechanisms involved in cell growth and cell invasiveness. These data provide a proof of principle to the hypothesis that the commonly hypomethylated genes in invasive cells could serve as a source for discovering new targets for inhibition of cancer cell invasiveness.

## DISCUSSION

Conversion of non-invasive cancer cells to invasive cells is a critical step in metastasis that alters the course and morbidity of the disease. Breast cancer as well as other cancer types are heterogeneous and include many cell subtypes with clear variances in gene expression programs and DNA methylation signatures [34–36]. In spite of this tremendous heterogeneity, invasive cancers that originated from different tissues share common fundamental biological mechanisms. These common mechanisms could provide broad-spectrum diagnostic tools and therapeutic targets. Since several of these mechanisms might be epigenetically controlled we developed an approach to identify common DNA methylation signatures of cancer invasiveness. We previously used DNA hypomethylation signatures to identify new target genes in hepatocellular carcinoma [37]. We reasoned that if such a common signature of invasiveness exists, it should be revealed by comparing human metastatic cancer cell lines from different origins to their non-invasive counterparts. Contrary to tumor specimens, cell lines contain a single cell type and could potentially reveal relatively homogenous and unconfounded DNA methylation profiles of highly invasive cells that would not be revealed in DNA methylation profiles of tumor specimens. We realized that cells in culture will acquire DNA methylation signatures that could be driven by stochastic events under culture conditions and that cells derived from different tissue origins will have different patterns of methylation. However, we reasoned that since these different cell lines exhibit a common phenotype, invasiveness, they should share common pathways that drive this phenotype through similar changes in DNA methylation. Such overlapping changes in different cell lines from different cellular origins and history in culture are highly unlikely to be caused by idiosyncrasies of the cell lines but most probably reflect the fundamental processes of invasiveness. Thus, the strategy utilized in this study effectively subtracts the background DNA methylation patterns associated with tissue specificity and culture conditions as well as growth and apoptosis to deliver an “invasion specific” signature. In addition, in contrast to cell growth programming that is under significant selection pressure in culture, there is no reason to believe that there is any selection pressure for pro-invasive genes or invasive phenotypes. Indeed our data suggest that the genes selected by our approach are not required for cell growth (Figure 6) and thus don't confer growth advantage and therefore were probably not artificially “selected” under the culture conditions.

Our analysis revealed a strong highly significant common DNA methylation signature that differentiates invasive from non-invasive cancer cells in spite of their vastly different tissues of origin. Two main lines of evidence suggest that these signatures are closely relevant to cancer metastasis. First, a pathway analysis performed by IPA reveals that many of the hypomethylated genes are members of functional gene pathways with strong experimental and clinical relevance to cancer. For example, molecular cellular functions include the following pathways, cellular growth and proliferation (191 genes out of 542 hypomethylated genes in invasive cancer cell lines; *p*-value range of 5.40E-14 - 4.22E-03) and cellular movement (128 genes out of 542; *p*-value range of 5.18E-13 - 4.03E-03). Second, we found a strong overlap between DNA hypomethylation of 5′UTR in metastatic cancer cell lines and induction of expression in clinical metastatic cancer samples which supports the functional role for DNA hypomethylation in cancer metastasis. Third, the hypomethylated genes in our signature are downstream to upstream regulators that have been implicated in cancer metastasis in numerous different studies (e.g., TGFB1, ERBB2). Fourth, knockdown of four genes from the list of hypomethylated genes, that were not assigned a role in metastasis to date, resulted in inhibition of invasiveness *in vitro* in at least one of three tested invasive cell types. Fifth, immunochemistry in clinical cancer samples and normal tissues reveals differential expression of three of the proteins in either breast, liver or prostate cancer (Table 2). Although all three tested proteins clearly differentiate all tested invasive cell lines from their non-invasive counterparts, the situation *in vivo* is more complex as expected due to heterogeneity of tumor biopsies.

**Table 2:** Summary of analysis of C11orf68, SHISA2 and TMEM156 expression in tissue arrays

Gene	Tissue		# of Patients and Healthy individuals	SEX	Age mean ± SD	Staining intensity Average ± SD	*P*-value
C11orf68	Breast	N/NAT	8	F	37 ± 11	0	-
		IDC/ILC	75	F	49 ± 10	3.4 ± 3	0.0039 **
		MC	9	F	49 ± 12	0.9 ± 2	0.2357
	Prostate	N/NAT	7	M	37 ± 5	0	-
		AC/LGML	80	M	67 ± 10	1.3 ± 2.2	0.145
SHISA2	Breast	N/NAT	3	F	44 ± 2	1 ± 1.7	-
		IDC/ILC	59	F	48 ± 10	5.6 ± 3	0.012 *
		MC	9	F	49.5 ± 12	4.9 ± 3.3	0.085
	Liver	N	17	F-6, M-11	34.2 ± 15	8.9 ± 1.2	-
		NAT	13	F-6, M-7	41.4 ± 15.6	5.7 ± 1.8	-
		HC	39	F-7, M-32	49.6 ± 10.7	7.9 ± 2.4	NAT = 0.001^1^ ***/*N* = 0.06^2^
	Prostate	N	3	M	34.6 ± 3	0	-
		AC	56	M	67.4 ± 11	4.2 ± 3	0.0197 *
TMEM156	Breast	N	8	F	30 ± 11.8	2.6 ± 3.6	-
		IDC/ILC	16	F	49.5 ± 7.4	8.9 ± 1.2	< 0.00001****
	Liver	N	8	F-4, M-4	46.5 ± 3.8	0	-
		HC	15	M	57 ± 5.5	6.7 ± 4.3	0.0003 ***
	Prostate	N	4	M	47 ± 15	5.5 ± 2.3	-
		AC/LGML	20	M	67 ± 4	7.05 ± 3.4	0.38

N - normal tissues from healthy individuals; NAT - cancer adjacent normal tissues; MC - breast medullary carcinoma; IDC - invasive ductal carcinoma; ILC-invasive lobular carcinoma; HC liver hepatocellular carcinoma; PAC - prostate adenocarcinoma; LGML - low grade malignant leiomyosarcoma.

Average staining intensity (combined out of staining intensity from 0 to 3 (negative to strong) and percentage of cells showing positive staining scored from 0 to 7), represent mean ± S.D of all samples score. Significant differences (p value) between cancer samples (IDC/ILC, MC, HC, AC, AC/LGML) and normal (N and NAT) are indicated by stars, indicating the level of efficiency:

**** *P* < 0.00001

*** *P* < 0.001

** *P* < 0.01

* *P* < 0.05.

1 *p*-value was calculated between HC and NAT

2 *p*-value was calculated between HC and N

The four genes that were examined in detail have not been previously reported to play a role in metastasis although two of these genes exhibit activities that suggest a role in invasiveness. *G0S2*, a G0/G1switch 2 protein, was recently reported to be a novel pro-apoptotic factor, which is induced upon tumor necrosis factor alpha (TNFα) treatment, whose activation also required NF-κB. G0S2 promotes apoptosis by interacting with Bcl-2 and by preventing the formation of protective Bcl-2/Bax heterodimers [29]. Moreover, *G0S2* was reported to be regulated by DNA methylation (hypermethylation) in squamous lung carcinoma [38] and to exhibit anti-proliferative activity in K562 erythroleukemia cells [39]. By contrast, we observe significant *G0S2* hypomethylation and induction in breast, liver and prostate invasive cancer cell lines (Figure 4D) that suggests the opposite role for this protein in these three cancer types.

*SHISA2* plays an essential role in the maturation of presomitic mesoderm cells by attenuation of both FGF and WNT signaling [33]. *SHISA2* was reported recently to be overexpressed in high-grade prostate cancer cells and to be involved in aggressive phenotype of prostate cancer [40]. Interestingly, CpG island within *SHISA2* promoter has also been reported to be hypomethylated in recurrent tumors in comparison with non-recurrent tumors [41]. SHISA2 staining in prostate adenocarcinoma confirms previous observations of *SHISA2* activation in prostate aggressive cancer [40]. The other genes that were examined, *TMEM156* and *C11orf68*, to our knowledge were never shown to have biochemical or cellular role that could be linked to cancer.

Our findings show the relevance of hypomethylation in metastatic cancer, which can have important therapeutic implications. Most of the past literature in the field of cancer focused on identifying methylated and silenced genes. However, silenced genes that suppress cancer could only be activated by epigenetic manipulations but these are not specific to the silenced genes and exert general effects such as inducing cancer-promoting genes. Our strategy identifies genes that are reprogrammed by hypomethylation and activated in metastatic cancer. Specific inhibitors could block the proteins encoded by these genes. These would target metastatic cancer and thus serve as novel drug targets in cancer metastasis treatment. The strategy applied in this manuscript could therefore allow the identification of common diagnostics of metastatic cancer, common mechanisms, as well as common drug targets. However, it is clear that these need to be tested in clinical samples as the complexities of tumor progression *in vivo* might result in altered role for such proteins as seen in our study and only a subset of these candidates would evolve to become broad scope targets in metastatic cancer.

## MATERIALS AND METHODS

### Cell lines and culture conditions

We used the following non-invasive cell lines: HepG2 [42] epithelial liver carcinoma derived from 15 years Caucasian male (#HB-8065), MCF7 [43] epithelial mammary gland adenocarcinoma derived from 69 years Caucasian female (#HTB-22), LNCaP [44] epithelial prostate adenocarcinoma derived from 62 years Caucasian male (#CRL 1740) and their invasive counterparts: SKHep1 [45] epithelial liver adenocarcinoma derived from 52 years Caucasian male (#HTB-52), MDA-MB-231 [46] epithelial mammary gland adenocarcinoma derived from 40 years Caucasian female (#HTB-231) and PC3 [47] epithelial prostate carcinoma derived from 50 years Caucasian male (#CRL-1435) which were purchased from ATCC, USA.

HepG2, SKHep1 and MCF-7 cells were maintained in MEM medium (Gibco, Invitrogen, Life Technologies). MCF7 cells were cultured with 0.01 mg/ml of insulin (Invitrogen, Carlsbad, California). MDA-MB-231 cells were cultured in Dulbecco's modified Eagle's medium (Invitrogen). PC3 and LNCaP were cultured in RPMI1640 media (Gibco, Invitrogen, Life Technologies). All media were supplemented with 2 mmol/L glutamine (Sigma-Aldrich), 10% FBS (Gibco), 1 U/mL penicillin and 1 mg/mL streptomycin (Gibco).

All cell lines were routinely verified by morphology and growth rate. HepG2, SKHep1, MCF7, MDA-MB-231 and LNCaP cells were authenticated by DNA profiling using the short tandem repeat, in 2014. PC-3 cells were authenticated by DNA testing in February 2013 and tested for MAP by PCR in March 2008.

### *In vitro* Invasion assay

Boyden chamber Matrigel Cell invasion assays were performed following the manufacturers protocol (Chemicon, Billerica, MA). Briefly, 3 × 10^5^ cells in serum-free media were plated for each treatment condition in the upper chamber containing the Matrigel-coated membranes. Serum-containing media acted as chemo-attractants in the lower chambers. After incubation for 48 h, the invaded cells at the bottom of the membrane were stained and counted under a light microscope with X400 magnification. All experiments were performed in triplicates.

### Trypan blue cell viability assay

Cell viability was determined by the Trypan blue (Sigma-Aldrich) exclusion test. ShRNAs treated cells were plated in 24-well plates in triplicate. After 48 hours cells were trypsinized and stained with trypan blue. Viable and non-viable cells were counted under a light microscope. The fraction of viable cell was determined by dividing live cells (non-stained) by total cells (both trypan blue positive and negative)

### Illumina 450K whole genome analysis

Genomic DNA was quantified using Picogreen protocol (Quant-iT™ PicoGreen^®^ dsDNA Products, Invitrogen, P-7589) and read on SpectraMAXGeminiXS Spectrophotometer. Bisulfate conversion was performed with 500 ng of genomic DNA using the EZ-96 DNA Methylation-Gold Kit (Zymo Research, D5007).

The Illumina Methylation 450K kit was used for the microarray experiment, as described by the manufacturer's protocol, except that 8μl of bisulfate converted material was utilized to initiate the amplification step. Hybridization and scanning was performed at the Genome Quebec Center.

Data analysis was performed with the Methylation module (version 1.9.0) of the GenomeStudio software (Illumina; version 2011.1) usingHumanMethylation450_15017482_v.1.2.bpm manifest.

### Illumina HT12 expression bead array

Total RNA from MCF7, MDA-MB-231, HepG2 and SKHep1cell lines was quantified using a NanoDrop Spectrophotometer ND-1000 (NanoDrop Technologies, Inc.) and its integrity was assessed using a 2100 Bioanalyzer (Agilent Technologies). Double stranded cDNA was synthesized from (250ng) of total RNA, and *in vitro* transcription was performed to produce biotin-labeled cRNA using Illumina^®^ TotalPrep RNA Amplification Kit, according to manufacturer's instructions (Life Technologies). The labeled cRNA was then normalized at (250ng) and hybridized on (Human HT-12_v4 arrays) according to Illumina's Whole-Genome Gene Expression Direct Hybridization Assay Guide. The BeadChips were incubated in an Ilumina Hybridization oven at 58°C for 14 to 20 hours at a rocking speed of 5. Beadchips were washed also according to Illumina's Whole-Genome Gene Expression Direct Hybridization Assay Guide and scanned on an Illumina iScan Reader.

**Protein extraction, Western blot** analysis were performed as described before [37]. Polyclonal antibodies to C11orf68, SHISA2 and TMEM156 were purchased from Abcam (ab103656, ab107724, ab122047 consequently), for G0S2 from USBiological (G8577-80A). Monoclonal antibodies to β-actin and α-Tubulin were purchased from Sigma Aldrich Co. (Cat#A5316, #T9026 consequently). Anti rabbit secondary antibody was purchased from Sigma Aldrich Co. (Cat#A0545) and anti mouse from Amersham Biosciences (Uppsala, Sweden) (final antibody concentration was according to the manufacturer's recommendation).

### shRNA Treatment

To suppress *C11ORF68, G0S2, SHISA2* and *TMEM156* genes we used lentivirus-mediated human pGIPZ shRNA plasmids and control pGIPZ-scrambled shRNA (Open Biosystems) (Supplementary Table S1). Lentiviruses were assembled using the following three vectors: GFP expression pGIPZ transfer vector—includes the insert (Open Biosystems); pMD2.G (VSV-G envelope expressing plasmid); PAX (packaging plasmid). The day before transfection, 10^6^ HEK293T cells were plated in a 10 cm dish (20–30% confluence). Next day, 5 μg of each vector were transfected using FuGene HD transfection reagent (Roche) according to the manufacturer's protocol. Cells were incubated for 48 h followed by collection of the medium containing the virus, filtered and used to infect the target cells. Selection with 1 mg/ml puromycin (Sigma) was started after 48 h postinfection. The rationale for using one of the shRNAs was based on knockdown efficiency in the specific cell line. The following (most efficient) shRNAs knockdowns were used for downstream experiments: *C11orf68* in MDA-MB-231 was targeted with ShC11orf68#5 and ShC11orf68#6, in SKHep1 with ShC11orf68#6 and ShC11orf68#7 and in PC3 with ShC11orf68#8. *G0S2 in* MDA-MB-231was targeted with ShG0S2#6, in SKHep1with ShG0S2#6 and ShG0S2#7 and in PC3 with ShG0S2#6. *SHISA2* in MDA-MB-231 was targeted with ShSHISA2#3 in SKHep1 with ShSHISA2#2 and ShSHISA2#3 and in PC3 with ShSHISA2#3. *TMEM156* in MDA-MB-231 was targeted with ShTMEM#10, in SKHep1 and in PC3 with ShTMEM156#3 (please see Table S1 for sequences)

### Statistical and bioinformatics method

GenomeStudio (V2011.1) Methylation module (1.9.0) and Expression module (1.9.0) software was used to generate heatmaps and perform statistical analysis for Illumina 450K methylation BeadChip array and Illumina HT12 BeadChip. Significant difference in methylation levels between invasive and non-invasive cancer cells was corrected for multiple testing using a False Discovery Rate (FDR) adjusted to *P* < 0.05. For expression analysis we used Quantile normalization, diffscore more than 13 or less than −13. At least two-fold differentially expressed genes were selected for further analysis.

The Ingenuity Pathway Analysis (IPA) program (http://www.ingenuity.com/index.html) was used to identify the potential affected gene networks, functional categories, canonical pathways and upstream regulators.

Heatmap on Figure 2 was created by the use of Gene E (http://www.broadinstitute.org/cancer/software/GENE-E/)

Statistical analysis of pyrosequencing, QPCR, invasion assay and cell viability assays was performed using an unpaired *t* test with two tailed distribution. The results were considered statistically significant when *P* < 0.05.

### RNA extraction, reverse transcription (RT) and quantitave polymerase chain reaction (QPCR)

Total RNA was isolated using TRIzol (Invitrogen, Life Technologies) according to the manufacturer's protocol. RT was performed using 1 μg of RNA and 20U avian myeloblastosis virus reverse transcriptase (Roche), as recommended by the manufacturer and QPCR was performed in a Roche LightCycler LC480. For quantification and normalization, the results were analyzed by the Roche LightCycler 480 software.

### DNA extraction, Bisulfate conversion and Pyrosequencing

DNA was extracted using standard phenol-chloroform protocol [48] and bisulfate conversion of genomic DNA was performed using the EZ DNA Methylation-Gold Kit (Zymo Research, D5005). Specific bisulfite converted sequences were amplified with HotStar Taq DNA polymerase (Qiagen) using biotinylated primers listed in Supplementary Table S1. The biotinylated DNA strands were pyrosequenced in the PyroMark Q24 instrument (Biotage, Qiagen) as previously described [49]. Data were analyzed using PyroMark Q24 software.

### Immunohistochemistry

Tissue Microarray slides were obtained from US Biomax Inc. (Rockville, MD). (Supplementary Tables S12, 13, 14 summarize the samples included in the arrays and the relevant clinical data). Rabbit polyclonal antibodies for C11orf68, SHISA2 and TMEM156 (Abcam, Toronto, ON) were used at 1:100, 1:50 and 1:20 dilution respectively. Heat-mediated antigen retrieval by citrate buffer at pH6 was performed for anti-TMEM156 and by Tris/EDTA pH 9.0 buffer, Envision™ FLEX Target Retrieval Solution (Dako, Burlington, ON) at 1:50 dilution for the rest of the antibodies (C11orf68, SHISA2). Phosphate buffer containing hydrogen peroxide, 15 mmol/L NaN3 and detergent, Envision™ FLEX Peroxidase-Blocking Reagent (Dako) was used as blocking reagent. As secondary antibodies, Dextran coupled with peroxidase molecules and goat secondary antibody molecules against rabbit immunoglobulins in buffered solution containing stabilizing protein and preservative (Envision™ FLEX/HRP, Dako) was used for 30 minutes. 3,3′-diaminobenzidine tetrachloride, Envision™ FLEX DAB+ Chromogen (Dako) and buffered solution containing hydrogen peroxide and preservative (Envision™ FLEX Substrate buffer, Dako) were added. Counterstaining was done with hematoxylin (1a Harris hematoxylin solution by MERCK KGaA, Darmstadt, Germany). After every step, sections were washed twice for 10 minutes in Tris buffered saline solution containing Tween 20, pH7.6, (Envision™ FLEX Wash Buffer, Dako) at 1:20 dilution. Mounting of the slides was done with DPX (MERCK KGaA).

Two pathologists independently scored all tissue array cores for the proportion and intensity of staining of tissues. A score of 0–3 was assigned to each core based on the intensity of staining from negative to strong, and score of 0–7 was given based on the percentage of cells showing positive staining. Both these scores were combined to get a total score.

## SUPPLEMENTAL TABLES












