# Supplementary material for: A common promoter hypomethylation signature in invasive breast, liver and prostate cancer cell lines reveals novel targets involved in cancer invasiveness
Source: Oncotarget. 2015 Sep 22;6(32):33253–68. doi: 10.18632/oncotarget.5291 (PMC4741763; doi:10.18632/oncotarget.5291)
Supplement: Supplementary file 1 [file oncotarget-06-33253-s001.pdf]

SUPPLEMENTAL TABLES

Supplementary Table S1: Primer sequences used for qPCR and in pyrosequencing. List of shRNA sequences used in the study

Primers used for QPCR

Genes	Accession #	Primer sequences for QPCR
**ANXA3**	NM_004039	For: 5′ CGGCCCAGCTTCCTTCAAAAT 3′<br>Rev: 5′ CCATATGCACTTGGGGGTGT3′
**C11ORF68**	NM_031450	For: 5′ TGAGGAGCTGGAAGAGGAGG 3′<br>Rev: 5′ CAAACACTAGCCAGGGGTCC 3′
**CD44**	NM_000610	For: 5′ AGCAACTGAGACAGCAACCA 3′<br>Rev: 5′ AGACGTACCAGCCATTTGTGT 3′
**G0S2**	NM_015714	For: 5′ GCCGTGCCACTAAGGTCATT 3′<br>Rev: 5′ GATCAGCTCCTGGACCGTTT 3′
**ELK3**	NM_005230	For: 5′ TTACAAGAGCCACAGACCGT 3′<br>Rev: 5′ TGCACTCTCCATACCCAGATG 3′
**FLNC**	NM_001458	For: 5′ GAGGAGGAGCCCTCTGAAGT 3′<br>Rev: 5′ CTCTGTGGCCCAGTGTGTG 3′
**HAS3**	NM_138612	For: 5′ ACTACATCCAGGTGTGCGAC 3′<br>Rev: 5′ TGGACATCTCCCCCGACTC 3′
**PLEC1**	NM_201380	For: 5′ AGGAGCTTGAGGAGGTGTCA 3′<br>Rev: 5′ CGATCCCGCTCATCTGTGG 3′
**Rab34**	NM_031934	For: 5′ GCCCTGAAGGAGAATGACCC 3′<br>Rev: 5′ AGCAGGGGTACTCAGATCCT 3′
**SHISA2**	NM_001007538	For: 5′ CTCGGCAGTGCCCATCTAC 3′<br>Rev: 5′ GCTTAGGCCGGAGACATCTG 3′
**TMEM156**	NM_024943	For: 5′ AGAGTGCAAAAGTGGCAGAGT 3′<br>Rev: 5′ TGCGTGGTCTCTGCTGAAAG 3′

Primer sequences used for pyrosequencing

Gene	Accession #	Primer sequences for pyrosequencing	Annealing temperature [°C]	Amplicon length [bp]
**ANXA3**	NM_004039	FW 5′- AGTTTGGGTTGGGAGGTTAA-3′<br>Rv Bio 5′-CCCCACCCCCCTCAAAACTACC<br>TACT- 3′ Seq 5′- GGGTTGGGAGGTTAAA-3′	55	155
**ELK3**	NM_005230	FW 5′- GGAATGATGGGGTTTTTTTATTGA<br>TA-3′ Rv Bio 5′- ACATCCTATACTCATATCCAC<br>TAACT- 3′ Seq 5′- GTTTTTAAGGTGTTGGTAG-3′	55	283
**PLEC1**	NM_201380	FW Bio 5′- GGGAGGGTAGGTAGGTTG-3′<br>Rv 5′- CTCTTAAAATACCCCCCACTC - 3′<br>Seq 5′- ACAATACCCACAAAAATTACAC -3′	55	116
**PLEC1**	NM_201380	FW 5′ - GGGAGGGTAGGTAGGTTG-3′<br>Rv Bio 5′- CTCTTAAAATACCCCCCACTC - 3′<br>Seq 5′- TGTAGGTTGTTAGTTTTTTTTTAGG -3′	55	116

Primer sequences used for pyrosequencing

Gene	Accession #	Primer sequences for pyrosequencing	Annealing temperature [°C]	Amplicon length [bp]
**C11orf68**	NM_031450	FW 5′ -GGTGTTTGGGTTTTTTTTGAGT-3′ Rv Bio 5′- AACCCCCTTATCCCCATCCATATAA TT- 3′ Seq 5′- GGATTTGGTGGTATTTGT-3′	52	
**G0S2**	NM_015714			
**SHISA2**	NM_001007538	FW Bio 5′ - GTTTTTTTGGGTATTTTTAAGATATGAAGT-3′ Rv Bio 5′- AACCCCCTTATCCCCATCCATATAATT- 3′ Seq 5′- GGATTTGGTGGTATTTGT-3′	52	
**TMEM156**	NM_024943	FW Bio 5′ - ATGATTTTAAGAAGTAAGGGGAT TT-3′ Rv Bio 5′- TCCTTCTACCTTAACCTCCCA AAATA - 3′ Seq 5′- CCACTTCCCCCAACCC-3′	52	

ShRNAs equences used for genes knockdown experiments:

List of shRNA sequences used in the study (Open Biosystems).

Catalog #	#	Mature Antisense
ShScr [RHS4346]		(non-silencing GIPZ lentiviral shRNAmir control; contains no homology to known mammalian genes)
ShC11orf68 [RHS4531-EG83638]	#5	V3LHS_306327 AGTCGTCCGTGTAAACACA
	#6	V3LHS_306330 AAACACAGATGACCTGGCG
	#7	V3LHS_306329 AGGTCAGTTCCACGTTGTT
	#8	V3LHS_306328 CTGAGTTGGGTGAACCGGG
ShG0S2 [RHS4531-EG50486]	#5	V3LHS_411679 TAGATAATTCACACAGTGT
	#6	V3LHS_381561 ACAGCTTCACCATCTTCCC
	#7	V3LHS_411678 TTCTCTGAAGTTAAGTCCA
ShSHISA2 [RHS4531-EG387914]	#2	V3LHS_392556 TCAGCACAGAGAAATTCGT
	#3	V3LHS_413361 TTTTTATCAGATCTAGGCA
	#4	V3LHS_413362 AAGATACAATCAAGAAGGG
ShTMEM156 [RHS4531-EG80008]	#3	V3LHS_344689 AGATTGCAAACACACTTCC
	#10	V2LHS_176950 ATCGATTTCTCCTTGCTTG

Supplementary Table S2: ***See Supplementary File S1.*** List of 2961 hypomethylated and 5368 hypermethylated CG sites and corresponding genes in all invasive (MDA-MB-231, SKHep1 and PC3) cancer cell lines when compared to non-invasive counterparts (MCF7, HepG2 and LNCaP). Target ID represents a single CpG locus in Illumina450K methylation bead array. dist-to-tss- indicated the distance from target CpG to transcription start site. Average beta value estimate of methylation level and delta beta is the differential methylation values between invasive and it's non-invasive counterpart

Supplementary Table S3: A. List of genes hypomethylated more than 25% with *P*-value < 0.05 in invasive cancer cell lines involved in epithelial-mesenchymal transition pathway.

#	Genes	Entrez Gene Name
1	NDRG1	N-myc downstream regulated 1
2	PLAU	plasminogen activator
3	PLAUR	plasminogen activator, urokinase receptor
4	SMAD3	SMAD family member 3
5	STAT3	signal transducer and activator of transcription 3 (acute-phase response factor)
6	STMN1	stathmin 1
7	THRB	thyroid hormone receptor, beta
8	VIM	Vimentin

B. Sixteen genes involved in TGF beta pathway out of 235 hypomethylated genes, which are not known to be involved in cancer or cancer metastasis

#	Symbol	Entrez Gene Name
1	ACSS1	acyl-CoA synthetase short-chain family member 1
2	AMOTL2	angiomotin like 2
3	CDC42EP3	CDC42 effector protein (Rho GTPase binding) 3
4	ECE1	endothelin converting enzyme 1
5	FXYD5	FXYD domain containing ion transport regulator 5
6	GNG7	guanine nucleotide binding protein (G protein), gamma 7
7	HAS3	hyaluronan synthase 3
8	HMOX2	heme oxygenase (decycling) 2
9	KLRD1	killer cell lectin-like receptor subfamily D, member 1
10	MAP4	microtubule-associated protein 4
11	MFAP2	microfibrillar-associated protein 2
12	MRPS6	mitochondrial ribosomal protein S6
13	NCAN	neurocan
14	PLS3	plastin 3
15	TRIM2	tripartite motif containing 2
16	ZMIZ1	zinc finger, MIZ-type containing 1

Supplementary Table S4: ***See Supplementary File S2.*** Hypomethylated in invasive cancer cells genes involved in movement, migration and invasion assessed by IPA analysis

Supplementary Table S5: List of differentially methylated genes in our study and publicly available DCIS and IBC methylation data

Common genes in our study and genes diff methylated in DCIS and IDC	Common genes in MCF7 vs. MDA in DCIS-IDC
PLAT	FAIM2
SCN2A	PPP2R1B
PDZK1	PLAT
MTMR7	SCN2A
C19orf77	PSG3
ZNF704	PEBP4
PLCH2	KHDC1L
PSG2	OPA3
ZAP70	PDZK1
CIB3	MTMR7
LOC284837	C5orf52
ZNF423	MYADML2
ITGA6	TRIM29
POU2AF1	MIR200B
COX4I2	LRRTM2
ADCY8	HMGCS2
GRIP1	HTRA3
HEXA	MED24
SESN3	ZNF704
CHRM5	FOXS1
ARSJ	PLCH2
HOXA3	SERPINA1
DENND5A	B3GNT1
GUCY1B2	HYAL2
LPAR3	CAPN8
PAM	ZNF154
	CASP4
	PSG2
	ZAP70
	FADS1
	C11orf52

(*Continued*)

Common genes in our study and genes diff methylated in DCIS and IDC	Common genes in MCF7 vs. MDA in DCIS-IDC
	SYCN
	LOC148696
	CIB3
	RNF212
	ASB10
	PACSIN2
	PNMAL2
	LOC284837
	CABP1
	PGLYRP2
	TGM5
	THAP4
	PRAF2
	USP35
	KCNK16
	CPA1
	DEFB135
	PHGR1
	GPRC5B
	AHR
	ZNF423
	FAM101A
	SERPINA11
	EGR1
	ITGA6
	C9orf152
	MCCC2
	ELN
	CUL7
	ATXN3L
	C4orf6
	POU2AF1
	TMPRSS13
	TM2D3
	COX4I2

(*Continued*)

Common genes in our study and genes diff methylated in DCIS and IDC	Common genes in MCF7 vs. MDA in DCIS-IDC
	RHOC
	GLRB
	PRRT1
	FAM83E
	ZBTB10
	ADCY8
	ATOH8
	BTBD10
	LMOD1
	KCTD14
	BEND6
	GRIP1
	TMEM26
	PGR
	SESN3
	FKBP1A
	DAXX
	PLOD1
	SPEN
	CHRM5
	TFF3
	TRAF5
	ICAM2
	ANKK1
	ARSJ
	HOXA3
	SLC6A9
	HPGDS
	MSL3
	CPA2
	CARS
	TELO2
	P4HA3
	DENND5A
	FAM198B

(*Continued*)

Common genes in our study and genes diff methylated in DCIS and IDC	Common genes in MCF7 vs. MDA in DCIS-IDC
	GUCY1B2
	MMP20
	ZNF776
	LPAR3
	LMAN2
	PGPEP1
	VPS18
	PAM
	APOBEC4
	HYALP1
	KRT5
	PLG
	STAP2

Supplementary Table S6: ***See Supplementary File S3*****.** Common genes in hypomethylated in invasive cancer cell lines: MDA-MB-231, SKHep1 and PC3 and in breast, liver and prostate invasive tissues consequently

Supplementary Table S7: ***See Supplementary File S4*****.** List of the genes, hypomethylated and induced in invasive cancer cell lines

Supplementary Table S8: ***See Supplementary File S5*****.** Immunohistochemistry analysis for C11orf68, G0S2, SHISA2 and TMEM156 expression in breast, liver and prostate cancer
