# Supplementary material for: A common promoter hypomethylation signature in invasive breast, liver and prostate cancer cell lines reveals novel targets involved in cancer invasiveness
Source: Oncotarget. 2015 Sep 22;6(32):33253–68. doi: 10.18632/oncotarget.5291 (PMC4741763; doi:10.18632/oncotarget.5291)
Supplement: Supplementary file 5 [file oncotarget-06-33253-s005.docx]

Table S7 List of the genes, hypomethylated and induced in invasive cancer cell lines

| **Genes** | **Describtion** | **Refseq** | **Metastasis** | **Target ID (Illumina 450K)** | **UCSC_REFGENE_GROUP** | **RELATION_TO_UCSC_CPG_ISLAND** |
| --- | --- | --- | --- | --- | --- | --- |
| ADM | adrenomedullin | NM_001124 | yes | cg05727225, cg06875754 | Body; 3'UTR | island, island |
| ANXA2 | annexin A2 pseudogene 3; annexin A2; annexin A2 pseudogene 1 | NM_001002857 | yes | cg11421255; cg20118643; cg22502206; cg06738887; cg11681321^Enh^; cg13313836 | TSS1500; TSS1500; TSS1500; 5'UTR; 5'UTR; 5'UTR | S_Shore; Island; Island;****;N_Shore; N_Shelf |
|  |  | NM_001002858 |  |  | TSS1500; TSS1500; TSS1500; Body; Body; Body |  |
| ARAP3 | ArfGAP with RhoGAP domain, ankyrin repeat and PH domain 3 | NM_022481 | yes | cg11136562^Enh^ | 5'UTR |  |
| ASAP2 | ArfGAP with SH3 domain, ankyrin repeat and PH domain 2 | NM_003887 | no | (cg05088820; cg11444072; cg19588016; cg23902076; cg26168881)^Enh^ | body X5 | N_Shelf |
| AXL | AXL receptor tyrosine kinase | NM_021913 | yes | cg27579501^DHS^ |  | N_Shelf |
| C11orf68 | chromosome 11 open reading frame 68 | NM_031450 | no | cg25422051; cg10467098 | 3'UTR; TSS150 | N_Shore; S_Shore |
| CAPN2 | calpain 2, (m/II) large subunit | NM_001748 | yes | cg02923485^Enh, DHS^ | Body |  |
| CAV2 | caveolin 2 | NM_001233 | no | cg12739419DHS; cg16260298DHS; cg25274503DHS | Body; Body; Body | S_Shore; Island; Island |
| CD44 | CD44 molecule (Indian blood group) | NM_001001391, NM_001001392 | yes | cg04171808^Enh, DHS^; cg14082886; cg23186333 | Body; Body; Body | ****; S_Shelf; S_Shelf |
| CD59 | CD59 molecule, complement regulatory protein | NM_000611 | no | cg23903301^Enh^ | 5'UTR |  |
| CHST7 | carbohydrate (N-acetylglucosamine 6-O) sulfotransferase 7 | NM_019886 | no | cg05695951 | 3'UTR | S_Shelf |
| CORO1C | coronin, actin binding protein, 1C | NM_014325 | no | cg09182455^Enh^ | 5'UTR |  |
| CYR61 | cysteine-rich, angiogenic inducer, 61 | NM_001554 | yes | cg07602008;cg13206932;cg15648041;cg17499345;cg20293609;cg22123915 | body;body,3'UTR,body,body,body | S_Shore |
| DNAJB6 | DnaJ (Hsp40) homolog, subfamily B, member 6 | NM_058246 | yes | (cg07993586; cg18245230)^Enh, DHS^ | Body; Body | N_shore, Island |
| DRAP1 | DR1-associated protein 1 (negative cofactor 2 alpha) | NM_006442 | no | cg10467098 | Body | S_Shore |
| EGFR | epidermal growth factor receptor (erythroblastic leukemia viral (v-erb-b) oncogene homolog, avian) | NM_005228 | yes | cg04625338^Enh, DHS^; cg11849717 | Body; Body | ****:Island |
| EHD4 | EH-domain containing 4 | NM_139265 | no | cg07202353^DHS^; cg22451412^Enh^ | Body; Body | N_Shore; ***** |
| EMP1 | epithelial membrane protein 1 | NM_001423 | no | cg14409083^DHS^ | 5'UTR;1stExon |  |
| ETS1 | v-ets erythroblastosis virus E26 oncogene homolog 1 (avian) | NM_005238 | yes | cg11588197; cg26503877; cg26559804 | Body; Body; Body | N_Shore; N_Shore; N_Shore |
| EXT1 | exostoses (multiple) 1 | NM_000127 | no | cg03276982^Enh^; cg11586249; cg11592677^Enh^; cg16286281 | Body; Body; Body; Body |  |
| FAM129A | family with sequence similarity 129, member A | NM_052966 | no | cg17464436^Enh^ | Body |  |
| FEZ2 | fasciculation and elongation protein zeta 2 (zygin II) | NM_005102 | no | cg22957691^Enh^ | Body |  |
| FHL2 | four and a half LIM domains 2 | NM_201555; NM_201557 | yes | cg00004700; cg02054792^DHS^ | 5'UTR; 5'UTR | N_Shore; Island |
| FJX1 | four jointed box 1 (Drosophila) | NM_014344 | yes | cg03483632 | TSS1500 | N_Shore |
| FLNC | filamin C, gamma (actin binding protein 280) | NM_001458 | yes | cg11590780; cg19730813 | TSS200; TSS1500 | N_Shore |
| FOSL1 | FOS-like antigen 1 | NM_005438 | yes | cg01672322 | 3'UTR | S_Shore |
| FXYD5 | FXYD domain containing ion transport regulator 5 | NM_014164, NM_144779 | yes | cg08543966 | 5'UTR; 5'UTR | S_Shore |
| G0S2 | G0/G1switch 2 | NM_015714 | no | (cg06616057; cg09666230^DHS^; cg14824901^DHS^; cg27176828^DHS^) ^Enh^ | TSS1500; TSS1500; TSS200; TSS200 | N_Shore; N_Shore; Island; Island |
| GNG12 | guanine nucleotide binding protein (G protein), gamma 12 | NM_018841 | no | cg02736560^Enh, DHS^ | 5'UTR |  |
| GSTP1 | glutathione S-transferase pi 1 | NM_000852 | no | (cg02659086; cg06928838; cg09038676; cg11566244; cg04920951; cg26250609)^DHS^ | TSS200; Body, Body; Body; 5'UTR; 5'UTR | Island |
| HMGA1 | hypothetical LOC100130009; high mobility group AT-hook 1 | NM_145899 | yes | cg00544436; cg03020951; cg18696576; cg25207224^Enh^; cg02654940; cg22797644 | TSS1500; TSS1500; TSS1500; Body; 5'UTR; 5'UTR | Island; Island; Island; ****; S_Shore; S_Shore |
| HPCAL1 | hippocalcin-like 1 | NM_134421 | no | cg00238897; cg16246488; cg18022012 | 5'UTR; 5'UTR; 5'UTR | Island; S_Shore; S_Shore |
| ITGA3 | [integrin, alpha 3 (antigen CD49C, alpha 3 subunit of VLA-3 receptor)](http://david.abcc.ncifcrf.gov/geneReportFull.jsp?rowids=787106) | NM_002204 | yes | cg13213536^Enh^; cg26184501^DHS^ | Body; Body | S_Shelf; **** |
| LAMC2 | [laminin, gamma 2](http://david.abcc.ncifcrf.gov/geneReportFull.jsp?rowids=801278) | NM_005562 | yes | cg22346461^Enh, DHS;^ cg23696949^Enh^; cg01949993^DHS^; cg04218899^DHS^; cg06211724^Enh, DHS^ | Body; Body; TSS200; 5'UTR; 5'UTR |  |
| LARP6 | [La ribonucleoprotein domain family, member 6](http://david.abcc.ncifcrf.gov/geneReportFull.jsp?rowids=794432) | NM_018357, NM_197958 | no | cg20645232^DHS^ | Body | N_Shore |
| MPRIP | [myosin phosphatase Rho interacting protein; similar to Myosin phosphatase Rho-interacting protein (Rho-interacting protein 3) (M-RIP) (RIP3) (p116Rip)](http://david.abcc.ncifcrf.gov/geneReportFull.jsp?rowids=814019) | NM_015134, NM_201274 | no | cg01890836^Enh, DHS^ | Body |  |
| MYEOV | [myeloma overexpressed (in a subset of t(11;14) positive multiple myelomas)](http://david.abcc.ncifcrf.gov/geneReportFull.jsp?rowids=808045) | NM_138768 | no | (cg01638792; cg24776407)^Enh, DHS^ | TSS1500; 5'UTR |  |
| NRP1 | [neuropilin 1](http://david.abcc.ncifcrf.gov/geneReportFull.jsp?rowids=822143) | NM_003873 | yes | cg25763127^Enh, DHS^ | Body |  |
| NT5E | [5'-nucleotidase, ecto (CD73)](http://david.abcc.ncifcrf.gov/geneReportFull.jsp?rowids=811418) | NM_002526 | no | cg06516476^Enh, DHS^; cg23172664^Enh^ | Body; Body |  |
| PKIA | [protein kinase (cAMP-dependent, catalytic) inhibitor alpha](http://david.abcc.ncifcrf.gov/geneReportFull.jsp?rowids=791043) | NM_006823, NM_181839 | no | cg09043127^DHS^ | 5'UTR; 5'UTR | Island |
| PLAT | plasminogen activator, tissue | NM_000930 | yes | cg00491021^Enh^ | 5'UTR |  |
| PLAUR | [plasminogen activator, urokinase receptor](http://david.abcc.ncifcrf.gov/geneReportFull.jsp?rowids=816149) | NM_001005376, NM_002659 | yes | cg17168630 | TSS1500; TSS1500 | S_Shore |
| PLEC1 | similar to Plectin 1 (PLTN) (PCN) (Hemidesmosomal protein 1) (HD1); plectin 1, intermediate filament binding protein 500kDa | NM_000445 | no | cg00253228; cg12864389; cg15628518; cg15628518; cg19893585; cg21672292; cg01753788; cg23934955; cg05696706; cg06045337; cg04255391^DHS^; cg07531549^DHS, Enh^; cg11147309^Enh^; cg13389508; cg14695663; cg17560015; cg25325005; cg23324953 | Body (all) | S_Shore; Island(5); N_Shelf; N_Shelf**; N_Shore; S_Shore; S_Shelf; N_Shelf(5)** |
| PLEK2 | pleckstrin 2 | NM_016445 | no | cg17641252 | Body | Island |
| PPP1R18 | protein phosphatase 1, regulatory subunit 18 | NM_133471 | no | cg00587922; cg01413582; cg07474957^DHS^; cg11288144^DHS^; cg13036352; cg23167351^DHS^; cg25659902; cg06644669 | Body, 1stExon; Body; Body; Body; Body; 1stExon; 5'UTR | N_Shelf; N_Shore; N_Shelf; ****; N_Shelf; N_Shelf; N_Shelf; N_Shore |
| PRDM8 | PR domain containing 8 | NM_020226 | no | cg06624978; cg19595012; cg26162932 | (TSS200; TSS200; TSS200)^DHS^ | S_Shore |
| PXDN | peroxidasin homolog (Drosophila) | NM_012293 | no | (cg08166568; cg08216099^Enh^; cg19517718)^DHS^ | Body; Body; Body | N_Shore; ****; Island |
| RAB34 | RAB34, member RAS oncogene family | NM_031934 | no | cg08839210^DHS^; cg21237418^DHS^; cg05668853; cg03452174^DHS^; cg19982230^DHS^; cg08032476^DHS^; cg22803868^DHS^; cg02530437^DHS^; cg12873610^DHS^; cg18686527^DHS^; cg21816330^DHS^ | TSS200; TSS200; Body; TSS1500; TSS1500; TSS1500; TSS1500; 5'UTR ; 5'UTR; 5'UTR; 5'UTR | Island(3); N_Shore(4); Islanf(4) |
| RAC2 | ras-related C3 botulinum toxin substrate 2 (rho family, small GTP binding protein Rac2) | NM_002872 | no | cg14072120; cg18265887 | 1stExon; Body |  |
| RAI14 | retinoic acid induced 14 | NM_015577 | no | cg03980224^Enh^ | 5'UTR |  |
| RIN2 | [Ras and Rab interactor 2](http://david.abcc.ncifcrf.gov/geneReportFull.jsp?rowids=786118) | NM_018993 | no | (cg03894068; cg12049875; cg16606773; cg19327615)^DHS^ | Body (4) | Island(3); N_Shore |
| S100A16 | S100 calcium binding protein A16 | NM_080388 | no | cg23851011 | TSS1500 |  |
| S100A6 | S100 calcium binding protein A6 | NM_014624 | yes | cg04998420^DHS^ | 5'UTR |  |
| SGCE | [sarcoglycan, epsilon](http://david.abcc.ncifcrf.gov/geneReportFull.jsp?rowids=789102) | NM_001099400 | no | cg04010684; cg08172445; cg15846744; cg16956426; cg17890778; cg23498273; cg25485192; cg25524350; cg26179590; cg27492749; cg22579075^DHS, Enh^ | Body (11) | N_Shore; Island(4); N_Shore(2); Island; N_Shore; Island |
| SHISA2 | [shisa homolog 2 (Xenopus laevis)](http://david.abcc.ncifcrf.gov/geneReportFull.jsp?rowids=806821) | NM_001007538 | no | cg06215691 | Body | N_Shore |
| SORBS3 | [sorbin and SH3 domain containing 3](http://david.abcc.ncifcrf.gov/geneReportFull.jsp?rowids=787828) | NM_005775 | no | (cg03572859; cg07459525; cg07896312; cg18530251; cg20568227; cg22321237)^DHS^ | 5'UTR; TSS200; TSS200; 5'UTR(3) | Island |
| SRGN | [serglycin](http://david.abcc.ncifcrf.gov/geneReportFull.jsp?rowids=780326) | NM_002727 | yes | cg17342283^DHS^ | 5'UTR |  |
| STX1A | [syntaxin 1A (brain)](http://david.abcc.ncifcrf.gov/geneReportFull.jsp?rowids=787836) | NM_004603 | no | cg01712428; cg02770724 | Body; Body | N_Shore; N_Shore |
| TAGLN2 | [transgelin 2](http://david.abcc.ncifcrf.gov/geneReportFull.jsp?rowids=790788) | NM_003564 | yes | cg16107628 | 5'UTR | N_Shore |
| TAP2 | [transporter 2, ATP-binding cassette, sub-family B (MDR/TAP)](http://david.abcc.ncifcrf.gov/geneReportFull.jsp?rowids=788029) | NM_000544 | no | cg03438552; cg08998192; cg12854186; cg22940798 | Body(4) | N_Shore(4) |
| TICAM2 | [transmembrane emp24 protein transport domain containing 7; toll-like receptor adaptor molecule 2](http://david.abcc.ncifcrf.gov/geneReportFull.jsp?rowids=781503) | NM_021649 | no | (cg01554060; cg22173752; cg08675743; cg08870042; cg19007249; cg20733436; cg21611170; cg24641522)^DHS^ | 1stExon; 1stExon; TSS1500; TSS200; TSS200; 5'UTR; TSS1500; 5'UTR | Island; Island; S_Shore; Island; S_Shore; Island; S_Shore; Island |
| TMEM156 | [transmembrane protein 156](http://david.abcc.ncifcrf.gov/geneReportFull.jsp?rowids=815383) | NM_024943 | no | cg25246082 | TSS1500 |  |
| TSPAN5 | [tetraspanin 5](http://david.abcc.ncifcrf.gov/geneReportFull.jsp?rowids=782064) | NM_005723 | no | cg18823647; cg22559669 | TSS1500; TSS1500 | S_Shore; Island |
| VCL | [vinculin](http://david.abcc.ncifcrf.gov/geneReportFull.jsp?rowids=822191) | NM_003373 | no | cg23434815^Enh^ | Body |  |
| VEGFC | [vascular endothelial growth factor C](http://david.abcc.ncifcrf.gov/geneReportFull.jsp?rowids=795422) | NM_005429 | yes | cg06679347^DHS^ | TSS1500 | Island |
| VIM | [vimentin](http://david.abcc.ncifcrf.gov/geneReportFull.jsp?rowids=814359) | NM_003380 | yes | cg01154046^DHS^; cg05151811^DHS^; cg15363487^DHS^; cg19111999^DHS^; cg20319091; cg26983469^DHS^; cg00146951^DHS^; cg08918274^DHS^; cg23912429^DHS^ | 5'UTR; TSS200; 5'UTR; TSS200; Body; 5'UTR(4) | Island; N_Shore; Island; N_Shore; Island; Island, N_Shore; N_Shore; Island |
| ZNF22 | [zinc finger protein 22 (KOX 15)](http://david.abcc.ncifcrf.gov/geneReportFull.jsp?rowids=773248) | NM_006963 | no | cg16512882^DHS^ | TSS200 | N_Shore |
| ZNF532 | [similar to zinc finger protein 347; zinc finger protein 532](http://david.abcc.ncifcrf.gov/geneReportFull.jsp?rowids=786974) | NM_018181 | no | cg03096126^DHS^; cg04212150; cg12737497^DHS^; cg12406559^DHS^ | 5'UTR(4) | Island(3); N_Shore |
