# Supplementary material for: A common promoter hypomethylation signature in invasive breast, liver and prostate cancer cell lines reveals novel targets involved in cancer invasiveness
Source: Oncotarget. 2015 Sep 22;6(32):33253–68. doi: 10.18632/oncotarget.5291 (PMC4741763; doi:10.18632/oncotarget.5291)
Supplement: Supplementary file 6 [file oncotarget-06-33253-s006.docx]

Table S8

Immunohistochemistry analysis for C11orf68, SHISA2 and TMEM156 expression in breast, liver and prostate cancer.

BREAST

| Array | Antibody | Pos | No. | Sex | Age | Organ | Pathology diagnosis | Grade | Stage | TNM | Type † | Proportion Score | Intensity Score | Total Score | Comments |
| --- | --- | --- | --- | --- | --- | --- | --- | --- | --- | --- | --- | --- | --- | --- | --- |
| T088A | C11orf68 | A1 | 1 | F | 55 | Breast | Invasive ductal carcinoma | 1 | IIb | T2N1M0 | Malignant | 7 | 2 | 9 |  |
| T088A | C11orf68 | A2 | 2 | F | 55 | Breast | Invasive ductal carcinoma | 1 | IIb | T2N1M0 | Malignant | 7 | 2 | 9 |  |
| T088A | C11orf68 | A3 | 3 | F | 39 | Breast | Invasive ductal carcinoma | 1 | IIIa | T2N2M0 | Malignant | 7 | 1 | 8 |  |
| T088A | C11orf68 | A4 | 4 | F | 39 | Breast | Invasive ductal carcinoma | 1 | IIIa | T2N2M0 | Malignant | 7 | 1 | 8 |  |
| T088A | C11orf68 | A5 | 5 | F | 55 | Breast | Invasive ductal carcinoma | 1 | IIb | T2N1M0 | Malignant | 7 | 1 | 8 |  |
| T088A | C11orf68 | A6 | 6 | F | 55 | Breast | Invasive ductal carcinoma | 1 | IIb | T2N1M0 | Malignant | 7 | 2 | 9 |  |
| T088A | C11orf68 | A7 | 7 | F | 39 | Breast | Invasive ductal carcinoma | 1 | IIIa | T2N2M0 | Malignant | 7 | 1 | 8 |  |
| T088A | C11orf68 | A8 | 8 | F | 39 | Breast | Invasive ductal carcinoma | 1 | IIIa | T2N2M0 | Malignant | 7 | 2 | 9 |  |
| T088A | C11orf68 | B1 | 9 | F | 57 | Breast | Invasive ductal carcinoma | 2 | IIa | T2N0M0 | Malignant | 2 | 1 | 3 |  |
| T088A | C11orf68 | B2 | 10 | F | 57 | Breast | Invasive ductal carcinoma | 2 | IIa | T2N0M0 | Malignant | 6 | 1 | 7 |  |
| T088A | C11orf68 | B3 | 11 | F | 47 | Breast | Invasive lobular carcinoma | – | IIa | T2N0M0 | Malignant | 6 | 1 | 7 |  |
| T088A | C11orf68 | B4 | 12 | F | 47 | Breast | Invasive lobular carcinoma | – | IIa | T2N0M0 | Malignant | 6 | 1 | 7 |  |
| T088A | C11orf68 | B5 | 13 | F | 57 | Breast | Invasive ductal carcinoma | 2 | IIa | T2N0M0 | Malignant | 7 | 1 | 8 |  |
| T088A | C11orf68 | B6 | 14 | F | 57 | Breast | Invasive ductal carcinoma | 2 | IIa | T2N0M0 | Malignant | 7 | 1 | 8 |  |
| T088A | C11orf68 | B7 | 15 | F | 47 | Breast | Invasive lobular carcinoma | – | IIa | T2N0M0 | Malignant | 4 | 1 | 5 |  |
| T088A | C11orf68 | B8 | 16 | F | 47 | Breast | Invasive lobular carcinoma | – | IIa | T2N0M0 | Malignant | 2 | 1 | 3 |  |
| T088A | C11orf68 | C1 | 17 | F | 41 | Breast | Cancer adjacent normal breast tissue | – | – | – | NAT | 0 | 0 | 0 |  |
| T088A | C11orf68 | C2 | 18 | F | 41 | Breast | Cancer adjacent normal breast tissue | – | – | – | NAT | 0 | 0 | 0 |  |
| T088A | C11orf68 | C3 | 19 | F | 19 | Breast | Normal breast tissue | – | – | – | Normal | 0 | 0 | 0 |  |
| T088A | C11orf68 | C4 | 20 | F | 19 | Breast | Normal breast tissue | – | – | – | Normal | na | na | na | No epithelial cells seen. |
| T088A | C11orf68 | C5 | 21 | F | 41 | Breast | Cancer adjacent normal breast tissue | – | – | – | NAT | 0 | 0 | 0 |  |
| T088A | C11orf68 | C6 | 22 | F | 41 | Breast | Cancer adjacent normal breast tissue | – | – | – | NAT | na | na | na | No epithelial cells seen. |
| T088A | C11orf68 | C7 | 23 | F | 19 | Breast | Normal breast tissue(fibrofatty tissue and blood vessel) | – | – | – | Normal | na | na | na | No epithelial cells seen. |
| T088A | C11orf68 | C8 | 24 | F | 19 | Breast | Normal breast tissue | – | – | – | Normal | 0 | 0 | 0 |  |
| T088A | C11orf68 | – | – | M | 58 | Skin | Malignant melanoma (tissue marker) | – |  |  | Malignant | 7 | 2 | 9 |  |
| BC08013a | C11orf68 | A1 | 1 | F | 47 | Breast | Invasive ductal carcinoma | 1 | IIA | T2N0M0 | Malignant | 0 | 0 | 0 |  |
| BC08013a | C11orf68 | A2 | 2 | F | 45 | Breast | Invasive ductal carcinoma | 1 | IIA | T2N0M0 | Malignant | 0 | 0 | 0 |  |
| BC08013a | C11orf68 | A3 | 3 | F | 38 | Breast | Invasive ductal carcinoma | 1 | IIB | T3N1M0 | Malignant | 0 | 0 | 0 |  |
| BC08013a | C11orf68 | A4 | 4 | F | 54 | Breast | Invasive ductal carcinoma | 1 | IIB | T3N0M0 | Malignant | 0 | 0 | 0 |  |
| BC08013a | C11orf68 | A5 | 5 | F | 59 | Breast | Invasive ductal carcinoma | 1 | IIIA | T2N2M0 | Malignant | 0 | 0 | 0 |  |
| BC08013a | C11orf68 | A6 | 6 | F | 44 | Breast | Invasive ductal carcinoma | 1 | IIIB | T4bN0M0 | Malignant | 0 | 0 | 0 |  |
| BC08013a | C11orf68 | A7 | 7 | F | 37 | Breast | Invasive ductal carcinoma | 2 | IIA | T2N0M0 | Malignant | 2 | 1 | 3 |  |
| BC08013a | C11orf68 | A8 | 8 | F | 53 | Breast | Invasive ductal carcinoma | 2 | IIB | T3N0M0 | Malignant | 0 | 0 | 0 |  |
| BC08013a | C11orf68 | A9 | 9 | F | 47 | Breast | Invasive ductal carcinoma | 2 | IIA | T2N0M0 | Malignant | 2 | 1 | 3 |  |
| BC08013a | C11orf68 | B1 | 10 | F | 66 | Breast | Invasive ductal carcinoma (sparse) | 1 | IIA | T2N0M0 | Malignant | 0 | 0 | 0 |  |
| BC08013a | C11orf68 | B2 | 11 | F | 79 | Breast | Invasive ductal carcinoma | 1 | IIIB | T4N0M0 | Malignant | 2 | 1 | 3 |  |
| BC08013a | C11orf68 | B3 | 12 | F | 74 | Breast | Invasive ductal carcinoma | 1 | IIIB | T4N0M0 | Malignant | 0 | 0 | 0 |  |
| BC08013a | C11orf68 | B4 | 13 | F | 48 | Breast | Invasive ductal carcinoma | 2 | IIIB | T4N2M0 | Malignant | 2 | 1 | 3 |  |
| BC08013a | C11orf68 | B5 | 14 | F | 50 | Breast | Invasive ductal carcinoma | 1 | IIA | T2N0M0 | Malignant | 0 | 0 | 0 |  |
| BC08013a | C11orf68 | B6 | 15 | F | 38 | Breast | Invasive ductal carcinoma | 1 | IIA | T2N0M0 | Malignant | 3 | 2 | 5 |  |
| BC08013a | C11orf68 | B7 | 16 | F | 38 | Breast | Invasive ductal carcinoma | 1 | IIA | T2N0M0 | Malignant | 0 | 0 | 0 |  |
| BC08013a | C11orf68 | B8 | 17 | F | 50 | Breast | Invasive ductal carcinoma | 2 | IIB | T2N1M0 | Malignant | 0 | 0 | 0 |  |
| BC08013a | C11orf68 | B9 | 18 | F | 49 | Breast | Invasive ductal carcinoma | 2 | IIIB | T4N1M0 | Malignant | 0 | 0 | 0 |  |
| BC08013a | C11orf68 | C1 | 19 | F | 45 | Breast | Invasive ductal carcinoma | 2 | I | T1N0M0 | Malignant | 0 | 0 | 0 |  |
| BC08013a | C11orf68 | C2 | 20 | F | 52 | Breast | Invasive ductal carcinoma | 3 | IIB | T2N1M0 | Malignant | 3 | 1 | 4 |  |
| BC08013a | C11orf68 | C3 | 21 | F | 53 | Breast | Invasive ductal carcinoma (sparse) | 1 | IIA | T2N0M0 | Malignant | 0 | 0 | 0 |  |
| BC08013a | C11orf68 | C4 | 22 | F | 28 | Breast | Invasive ductal carcinoma | 2 | IIA | T1N1M0 | Malignant | 6 | 1 | 7 |  |
| BC08013a | C11orf68 | C5 | 23 | F | 52 | Breast | Invasive ductal carcinoma | 2 | IIA | T2N0M0 | Malignant | 0 | 0 | 0 |  |
| BC08013a | C11orf68 | C6 | 24 | F | 53 | Breast | Invasive ductal carcinoma | 2 | IIA | T2N0M0 | Malignant | 2 | 1 | 3 |  |
| BC08013a | C11orf68 | C7 | 25 | F | 42 | Breast | Invasive ductal carcinoma | 2 | IIB | T3N0M0 | Malignant | 2 | 1 | 3 |  |
| BC08013a | C11orf68 | C8 | 26 | F | 50 | Breast | Invasive ductal carcinoma | 2 | IIA | T2N0M0 | Malignant | 2 | 1 | 3 |  |
| BC08013a | C11orf68 | C9 | 27 | F | 60 | Breast | Invasive ductal carcinoma | 2 | IIB | T3N0M0 | Malignant | 5 | 1 | 6 |  |
| BC08013a | C11orf68 | D1 | 28 | F | 48 | Breast | Invasive ductal carcinoma | 2 | IIIB | T4N0M0 | Malignant | 0 | 0 | 0 |  |
| BC08013a | C11orf68 | D2 | 29 | F | 52 | Breast | Invasive ductal carcinoma (fibrous tissue and blood vessel) | – | IIIA | T2N2M0 | Malignant | 1 | 1 | 2 |  |
| BC08013a | C11orf68 | D3 | 30 | F | 32 | Breast | Invasive ductal carcinoma | 2 | IIB | T2N1M0 | Malignant | 0 | 0 | 0 |  |
| BC08013a | C11orf68 | D4 | 31 | F | 51 | Breast | Invasive ductal carcinoma | 2 | IIIA | T3N1M0 | Malignant | 0 | 0 | 0 |  |
| BC08013a | C11orf68 | D5 | 32 | F | 46 | Breast | Invasive ductal carcinoma | 2 | IIA | T2N0M0 | Malignant | 7 | 2 | 9 |  |
| BC08013a | C11orf68 | D6 | 33 | F | 48 | Breast | Invasive ductal carcinoma | 2 | IIB | T2N1M0 | Malignant | 6 | 1 | 7 |  |
| BC08013a | C11orf68 | D7 | 34 | F | 38 | Breast | Invasive ductal carcinoma | 2 | IIB | T2N1M0 | Malignant | 3 | 1 | 4 |  |
| BC08013a | C11orf68 | D8 | 35 | F | 54 | Breast | Invasive ductal carcinoma | 2 | IIB | T2N1M0 | Malignant | 3 | 1 | 4 |  |
| BC08013a | C11orf68 | D9 | 36 | F | 55 | Breast | Invasive ductal carcinoma | 2 | IIIB | T4N1M0 | Malignant | 0 | 0 | 0 |  |
| BC08013a | C11orf68 | E1 | 37 | F | 38 | Breast | Invasive ductal carcinoma | 2 | IIB | T2N1M0 | Malignant | 1 | 1 | 2 |  |
| BC08013a | C11orf68 | E2 | 38 | F | 53 | Breast | Invasive ductal carcinoma | 2 | I | T1cN0M0 | Malignant | 2 | 1 | 3 |  |
| BC08013a | C11orf68 | E3 | 39 | F | 40 | Breast | Invasive ductal carcinoma | 2 | IIA | T2N0M0 | Malignant | 4 | 1 | 5 |  |
| BC08013a | C11orf68 | E4 | 40 | F | 40 | Breast | Invasive ductal carcinoma | 2 | IIB | T3N0M0 | Malignant | 2 | 1 | 3 |  |
| BC08013a | C11orf68 | E5 | 41 | F | 47 | Breast | Invasive ductal carcinoma (fibrofatty tissue) | – | IIB | T3N1M0 | Malignant | na | na | na | No epithelial cells seen. |
| BC08013a | C11orf68 | E6 | 42 | F | 57 | Breast | Invasive ductal carcinoma | 2 | IIIB | T4N2M0 | Malignant | 2 | 1 | 3 |  |
| BC08013a | C11orf68 | E7 | 43 | F | 42 | Breast | Invasive ductal carcinoma | 2 | IIA | T2N0M0 | Malignant | 0 | 0 | 0 |  |
| BC08013a | C11orf68 | E8 | 44 | F | 46 | Breast | Invasive ductal carcinoma (chronic inflammation) | – | I | T1N0M0 | Malignant | 6 | 1 | 7 |  |
| BC08013a | C11orf68 | E9 | 45 | F | 38 | Breast | Invasive ductal carcinoma with necrosis (sparse) | – | IIIB | T4N0M0 | Malignant | 0 | 0 | 0 |  |
| BC08013a | C11orf68 | F1 | 46 | F | 45 | Breast | Invasive ductal carcinoma | 2 | IIIB | T4N0M0 | Malignant | 2 | 1 | 3 |  |
| BC08013a | C11orf68 | F2 | 47 | F | 47 | Breast | Invasive ductal carcinoma | 2 | IIB | T3N0M0 | Malignant | 2 | 1 | 3 |  |
| BC08013a | C11orf68 | F3 | 48 | F | 47 | Breast | Invasive ductal carcinoma | 2 | IIIB | T4N0M0 | Malignant | 5 | 2 | 7 |  |
| BC08013a | C11orf68 | F4 | 49 | F | 50 | Breast | Invasive ductal carcinoma | 3 | IIIB | T4N0M0 | Malignant | 5 | 1 | 6 |  |
| BC08013a | C11orf68 | F5 | 50 | F | 50 | Breast | Invasive ductal carcinoma | 3 | IIB | T2N1M0 | Malignant | 0 | 0 | 0 |  |
| BC08013a | C11orf68 | F6 | 51 | F | 38 | Breast | Invasive ductal carcinoma | 1 | IIA | T2N0M0 | Malignant | 7 | 1 | 8 |  |
| BC08013a | C11orf68 | F7 | 52 | F | 37 | Breast | Invasive ductal carcinoma | 3 | IIB | T2N1M0 | Malignant | 0 | 0 | 0 |  |
| BC08013a | C11orf68 | F8 | 53 | F | 76 | Breast | Invasive ductal carcinoma | 3 | IIIB | T4N0M0 | Malignant | 5 | 1 | 6 |  |
| BC08013a | C11orf68 | F9 | 54 | F | 52 | Breast | Invasive ductal carcinoma | 3 | IIB | T3N0M0 | Malignant | 2 | 1 | 3 |  |
| BC08013a | C11orf68 | G1 | 55 | F | 55 | Breast | Invasive ductal carcinoma | 3 | IV | T2N1M1 | Malignant | 0 | 0 | 0 |  |
| BC08013a | C11orf68 | G2 | 56 | F | 38 | Breast | Invasive ductal carcinoma | 3 | IIB | T3N0M0 | Malignant | 5 | 2 | 7 |  |
| BC08013a | C11orf68 | G3 | 57 | F | 32 | Breast | Invasive ductal carcinoma | 3 | IIA | T1N1M0 | Malignant | 3 | 1 | 4 |  |
| BC08013a | C11orf68 | G4 | 58 | F | 62 | Breast | Invasive ductal carcinoma | 3 | IIB | T2N1M0 | Malignant | 0 | 0 | 0 |  |
| BC08013a | C11orf68 | G5 | 59 | F | 65 | Breast | Invasive ductal carcinoma | 3 | IIIA | T1N2M0 | Malignant | 0 | 0 | 0 |  |
| BC08013a | C11orf68 | G6 | 60 | F | 35 | Breast | Invasive ductal carcinoma | 3 | IIA | T2N0M0 | Malignant | 0 | 0 | 0 |  |
| BC08013a | C11orf68 | G7 | 61 | F | 58 | Breast | Medullary carcinoma | – | IIA | T2N0M0 | Malignant | 0 | 0 | 0 |  |
| BC08013a | C11orf68 | G8 | 62 | F | 72 | Breast | Medullary carcinoma (sparse) | – | IIA | T2N0M0 | Malignant | 5 | 1 | 6 |  |
| BC08013a | C11orf68 | G9 | 63 | F | 39 | Breast | Medullary carcinoma | – | IIB | T2N1M0 | Malignant | 1 | 1 | 2 |  |
| BC08013a | C11orf68 | H1 | 64 | F | 50 | Breast | Medullary carcinoma | – | IIB | T3N0M0 | Malignant | 0 | 0 | 0 |  |
| BC08013a | C11orf68 | H2 | 65 | F | 48 | Breast | Medullary carcinoma | – | IIIB | T4N0M0 | Malignant | 0 | 0 | 0 |  |
| BC08013a | C11orf68 | H3 | 66 | F | 40 | Breast | Medullary carcinoma | – | IIIA | T2N2M0 | Malignant | 0 | 0 | 0 |  |
| BC08013a | C11orf68 | H4 | 67 | F | 55 | Breast | Medullary carcinoma | – | IIA | T2N0M0 | Malignant | 0 | 0 | 0 |  |
| BC08013a | C11orf68 | H5 | 68 | F | 31 | Breast | Medullary carcinoma | – | IIA | T2N0M0 | Malignant | 0 | 0 | 0 |  |
| BC08013a | C11orf68 | H6 | 69 | F | 52 | Breast | Medullary carcinoma (sparse) | – | IIA | T2N0M0 | Malignant | 0 | 0 | 0 |  |
| BC08013a | C11orf68 | H7 | 70 | F | 46 | Breast | Cancer adjacent normal breast tissue | – | – | – | NAT | 0 | 0 | 0 |  |
| BC08013a | C11orf68 | H8 | 71 | F | 46 | Breast | Cancer adjacent normal breast tissue | – | – | – | NAT | 0 | 0 | 0 |  |
| BC08013a | C11orf68 | H9 | 72 | F | 42 | Breast | Cancer adjacent normal breast tissue | – | – | – | NAT | 0 | 0 | 0 |  |
| BC08013a | C11orf68 | – | – | M | 42 | Adrenal gland | Pheochromocytoma (tissue marker) | – |  |  | Malignant | 0 | 0 | 0 |  |
| BC08013a | SHISA2 | A1 | 1 | F | 47 | Breast | Invasive ductal carcinoma | 1 | IIA | T2N0M0 | Malignant | 7 | 2 | 9 |  |
| BC08013a | SHISA2 | A2 | 2 | F | 45 | Breast | Invasive ductal carcinoma | 1 | IIA | T2N0M0 | Malignant | 2 | 1 | 3 |  |
| BC08013a | SHISA2 | A3 | 3 | F | 38 | Breast | Invasive ductal carcinoma | 1 | IIB | T3N1M0 | Malignant | 4 | 1 | 5 |  |
| BC08013a | SHISA2 | A4 | 4 | F | 54 | Breast | Invasive ductal carcinoma | 1 | IIB | T3N0M0 | Malignant | 6 | 2 | 8 |  |
| BC08013a | SHISA2 | A5 | 5 | F | 59 | Breast | Invasive ductal carcinoma | 1 | IIIA | T2N2M0 | Malignant | 4 | 2 | 6 |  |
| BC08013a | SHISA2 | A6 | 6 | F | 44 | Breast | Invasive ductal carcinoma | 1 | IIIB | T4bN0M0 | Malignant | 6 | 2 | 8 |  |
| BC08013a | SHISA2 | A7 | 7 | F | 37 | Breast | Invasive ductal carcinoma | 2 | IIA | T2N0M0 | Malignant | 4 | 2 | 6 |  |
| BC08013a | SHISA2 | A8 | 8 | F | 53 | Breast | Invasive ductal carcinoma | 2 | IIB | T3N0M0 | Malignant | 2 | 1 | 3 |  |
| BC08013a | SHISA2 | A9 | 9 | F | 47 | Breast | Invasive ductal carcinoma | 2 | IIA | T2N0M0 | Malignant | 4 | 1 | 5 |  |
| BC08013a | SHISA2 | B1 | 10 | F | 66 | Breast | Invasive ductal carcinoma (sparse) | 1 | IIA | T2N0M0 | Malignant | 4 | 1 | 5 |  |
| BC08013a | SHISA2 | B2 | 11 | F | 79 | Breast | Invasive ductal carcinoma | 1 | IIIB | T4N0M0 | Malignant | 4 | 1 | 5 |  |
| BC08013a | SHISA2 | B3 | 12 | F | 74 | Breast | Invasive ductal carcinoma | 1 | IIIB | T4N0M0 | Malignant | 4 | 1 | 5 |  |
| BC08013a | SHISA2 | B4 | 13 | F | 48 | Breast | Invasive ductal carcinoma | 2 | IIIB | T4N2M0 | Malignant | 6 | 2 | 8 |  |
| BC08013a | SHISA2 | B5 | 14 | F | 50 | Breast | Invasive ductal carcinoma | 1 | IIA | T2N0M0 | Malignant | 3 | 1 | 4 |  |
| BC08013a | SHISA2 | B6 | 15 | F | 38 | Breast | Invasive ductal carcinoma | 1 | IIA | T2N0M0 | Malignant | 6 | 2 | 8 |  |
| BC08013a | SHISA2 | B7 | 16 | F | 38 | Breast | Invasive ductal carcinoma | 1 | IIA | T2N0M0 | Malignant | 6 | 2 | 8 |  |
| BC08013a | SHISA2 | B8 | 17 | F | 50 | Breast | Invasive ductal carcinoma | 2 | IIB | T2N1M0 | Malignant | 2 | 1 | 3 |  |
| BC08013a | SHISA2 | B9 | 18 | F | 49 | Breast | Invasive ductal carcinoma | 2 | IIIB | T4N1M0 | Malignant | 4 | 1 | 5 |  |
| BC08013a | SHISA2 | C1 | 19 | F | 45 | Breast | Invasive ductal carcinoma | 2 | I | T1N0M0 | Malignant | 6 | 2 | 8 |  |
| BC08013a | SHISA2 | C2 | 20 | F | 52 | Breast | Invasive ductal carcinoma | 3 | IIB | T2N1M0 | Malignant | 6 | 3 | 9 |  |
| BC08013a | SHISA2 | C3 | 21 | F | 53 | Breast | Invasive ductal carcinoma (sparse) | 1 | IIA | T2N0M0 | Malignant | 4 | 1 | 5 |  |
| BC08013a | SHISA2 | C4 | 22 | F | 28 | Breast | Invasive ductal carcinoma | 2 | IIA | T1N1M0 | Malignant | 6 | 1 | 7 |  |
| BC08013a | SHISA2 | C5 | 23 | F | 52 | Breast | Invasive ductal carcinoma | 2 | IIA | T2N0M0 | Malignant | 0 | 0 | 0 |  |
| BC08013a | SHISA2 | C6 | 24 | F | 53 | Breast | Invasive ductal carcinoma | 2 | IIA | T2N0M0 | Malignant | 2 | 1 | 3 |  |
| BC08013a | SHISA2 | C7 | 25 | F | 42 | Breast | Invasive ductal carcinoma | 2 | IIB | T3N0M0 | Malignant | 3 | 1 | 4 |  |
| BC08013a | SHISA2 | C8 | 26 | F | 50 | Breast | Invasive ductal carcinoma | 2 | IIA | T2N0M0 | Malignant | 3 | 2 | 5 |  |
| BC08013a | SHISA2 | C9 | 27 | F | 60 | Breast | Invasive ductal carcinoma | 2 | IIB | T3N0M0 | Malignant | 7 | 2 | 9 |  |
| BC08013a | SHISA2 | D1 | 28 | F | 48 | Breast | Invasive ductal carcinoma | 2 | IIIB | T4N0M0 | Malignant | 0 | 0 | 0 |  |
| BC08013a | SHISA2 | D2 | 29 | F | 52 | Breast | Invasive ductal carcinoma (fibrous tissue and blood vessel) | – | IIIA | T2N2M0 | Malignant | 7 | 2 | 9 |  |
| BC08013a | SHISA2 | D3 | 30 | F | 32 | Breast | Invasive ductal carcinoma | 2 | IIB | T2N1M0 | Malignant | 0 | 0 | 0 |  |
| BC08013a | SHISA2 | D4 | 31 | F | 51 | Breast | Invasive ductal carcinoma | 2 | IIIA | T3N1M0 | Malignant | 2 | 1 | 3 |  |
| BC08013a | SHISA2 | D5 | 32 | F | 46 | Breast | Invasive ductal carcinoma | 2 | IIA | T2N0M0 | Malignant | 7 | 3 | 10 |  |
| BC08013a | SHISA2 | D6 | 33 | F | 48 | Breast | Invasive ductal carcinoma | 2 | IIB | T2N1M0 | Malignant | 4 | 2 | 6 |  |
| BC08013a | SHISA2 | D7 | 34 | F | 38 | Breast | Invasive ductal carcinoma | 2 | IIB | T2N1M0 | Malignant | 7 | 2 | 9 |  |
| BC08013a | SHISA2 | D8 | 35 | F | 54 | Breast | Invasive ductal carcinoma | 2 | IIB | T2N1M0 | Malignant | 1 | 1 | 2 |  |
| BC08013a | SHISA2 | D9 | 36 | F | 55 | Breast | Invasive ductal carcinoma | 2 | IIIB | T4N1M0 | Malignant | 0 | 0 | 0 |  |
| BC08013a | SHISA2 | E1 | 37 | F | 38 | Breast | Invasive ductal carcinoma | 2 | IIB | T2N1M0 | Malignant | 2 | 1 | 3 |  |
| BC08013a | SHISA2 | E2 | 38 | F | 53 | Breast | Invasive ductal carcinoma | 2 | I | T1cN0M0 | Malignant | 7 | 2 | 9 |  |
| BC08013a | SHISA2 | E3 | 39 | F | 40 | Breast | Invasive ductal carcinoma | 2 | IIA | T2N0M0 | Malignant | 7 | 2 | 9 |  |
| BC08013a | SHISA2 | E4 | 40 | F | 40 | Breast | Invasive ductal carcinoma | 2 | IIB | T3N0M0 | Malignant | 7 | 2 | 9 |  |
| BC08013a | SHISA2 | E5 | 41 | F | 47 | Breast | Invasive ductal carcinoma (fibrofatty tissue) | – | IIB | T3N1M0 | Malignant | na | na | na | No tumor |
| BC08013a | SHISA2 | E6 | 42 | F | 57 | Breast | Invasive ductal carcinoma | 2 | IIIB | T4N2M0 | Malignant | 7 | 3 | 10 |  |
| BC08013a | SHISA2 | E7 | 43 | F | 42 | Breast | Invasive ductal carcinoma | 2 | IIA | T2N0M0 | Malignant | 4 | 1 | 5 |  |
| BC08013a | SHISA2 | E8 | 44 | F | 46 | Breast | Invasive ductal carcinoma (chronic inflammation) | – | I | T1N0M0 | Malignant | 7 | 2 | 9 |  |
| BC08013a | SHISA2 | E9 | 45 | F | 38 | Breast | Invasive ductal carcinoma with necrosis (sparse) | – | IIIB | T4N0M0 | Malignant | 0 | 0 | 0 |  |
| BC08013a | SHISA2 | F1 | 46 | F | 45 | Breast | Invasive ductal carcinoma | 2 | IIIB | T4N0M0 | Malignant | 6 | 1 | 7 |  |
| BC08013a | SHISA2 | F2 | 47 | F | 47 | Breast | Invasive ductal carcinoma | 2 | IIB | T3N0M0 | Malignant | 3 | 1 | 4 |  |
| BC08013a | SHISA2 | F3 | 48 | F | 47 | Breast | Invasive ductal carcinoma | 2 | IIIB | T4N0M0 | Malignant | 7 | 2 | 9 |  |
| BC08013a | SHISA2 | F4 | 49 | F | 50 | Breast | Invasive ductal carcinoma | 3 | IIIB | T4N0M0 | Malignant | 7 | 2 | 9 |  |
| BC08013a | SHISA2 | F5 | 50 | F | 50 | Breast | Invasive ductal carcinoma | 3 | IIB | T2N1M0 | Malignant | 6 | 1 | 7 |  |
| BC08013a | SHISA2 | F6 | 51 | F | 38 | Breast | Invasive ductal carcinoma | 1 | IIA | T2N0M0 | Malignant | 7 | 2 | 9 |  |
| BC08013a | SHISA2 | F7 | 52 | F | 37 | Breast | Invasive ductal carcinoma | 3 | IIB | T2N1M0 | Malignant | 4 | 1 | 5 |  |
| BC08013a | SHISA2 | F8 | 53 | F | 76 | Breast | Invasive ductal carcinoma | 3 | IIIB | T4N0M0 | Malignant | 2 | 1 | 3 |  |
| BC08013a | SHISA2 | F9 | 54 | F | 52 | Breast | Invasive ductal carcinoma | 3 | IIB | T3N0M0 | Malignant | 6 | 2 | 8 |  |
| BC08013a | SHISA2 | G1 | 55 | F | 55 | Breast | Invasive ductal carcinoma | 3 | IV | T2N1M1 | Malignant | 0 | 0 | 0 |  |
| BC08013a | SHISA2 | G2 | 56 | F | 38 | Breast | Invasive ductal carcinoma | 3 | IIB | T3N0M0 | Malignant | 7 | 2 | 9 |  |
| BC08013a | SHISA2 | G3 | 57 | F | 32 | Breast | Invasive ductal carcinoma | 3 | IIA | T1N1M0 | Malignant | 6 | 2 | 8 |  |
| BC08013a | SHISA2 | G4 | 58 | F | 62 | Breast | Invasive ductal carcinoma | 3 | IIB | T2N1M0 | Malignant | 1 | 1 | 2 |  |
| BC08013a | SHISA2 | G5 | 59 | F | 65 | Breast | Invasive ductal carcinoma | 3 | IIIA | T1N2M0 | Malignant | 0 | 0 | 0 |  |
| BC08013a | SHISA2 | G6 | 60 | F | 35 | Breast | Invasive ductal carcinoma | 3 | IIA | T2N0M0 | Malignant | 4 | 2 | 6 |  |
| BC08013a | SHISA2 | G7 | 61 | F | 58 | Breast | Medullary carcinoma | – | IIA | T2N0M0 | Malignant | 5 | 1 | 6 |  |
| BC08013a | SHISA2 | G8 | 62 | F | 72 | Breast | Medullary carcinoma (sparse) | – | IIA | T2N0M0 | Malignant | 0 | 0 | 0 |  |
| BC08013a | SHISA2 | G9 | 63 | F | 39 | Breast | Medullary carcinoma | – | IIB | T2N1M0 | Malignant | 0 | 0 | 0 |  |
| BC08013a | SHISA2 | H1 | 64 | F | 50 | Breast | Medullary carcinoma | – | IIB | T3N0M0 | Malignant | 5 | 1 | 6 |  |
| BC08013a | SHISA2 | H2 | 65 | F | 48 | Breast | Medullary carcinoma | – | IIIB | T4N0M0 | Malignant | 3 | 1 | 4 |  |
| BC08013a | SHISA2 | H3 | 66 | F | 40 | Breast | Medullary carcinoma | – | IIIA | T2N2M0 | Malignant | 3 | 1 | 4 |  |
| BC08013a | SHISA2 | H4 | 67 | F | 55 | Breast | Medullary carcinoma | – | IIA | T2N0M0 | Malignant | 7 | 2 | 9 |  |
| BC08013a | SHISA2 | H5 | 68 | F | 31 | Breast | Medullary carcinoma | – | IIA | T2N0M0 | Malignant | 7 | 2 | 9 |  |
| BC08013a | SHISA2 | H6 | 69 | F | 52 | Breast | Medullary carcinoma (sparse) | – | IIA | T2N0M0 | Malignant | 5 | 1 | 6 |  |
| BC08013a | SHISA2 | H7 | 70 | F | 46 | Breast | Cancer adjacent normal breast tissue | – | – | – | NAT | 2 | 1 | 3 |  |
| BC08013a | SHISA2 | H8 | 71 | F | 46 | Breast | Cancer adjacent normal breast tissue | – | – | – | NAT | 0 | 0 | 0 |  |
| BC08013a | SHISA2 | H9 | 72 | F | 42 | Breast | Cancer adjacent normal breast tissue | – | – | – | NAT | 0 | 0 | 0 |  |
| BC08013a | SHISA2 | – | – | M | 42 | Adrenal gland | Pheochromocytoma (tissue marker) | – |  |  | Malignant | 0 | 0 | 0 |  |
| T088a | TMEM156 | – | – | M | 58 | Skin | Malignant melanoma (tissue marker) | – |  |  | Malignant | 0 | 0 | 0 |  |
| T088a | TMEM156 | A1 | 1 | F | 55 | Breast | Invasive ductal carcinoma | 1 | IIb | T2N1M0 | Malignant | 7 | 2 | 9 |  |
| T088a | TMEM156 | A2 | 2 | F | 55 | Breast | Invasive ductal carcinoma | 1 | IIb | T2N1M0 | Malignant | 7 | 3 | 10 |  |
| T088a | TMEM156 | A3 | 3 | F | 39 | Breast | Invasive ductal carcinoma | 1 | IIIa | T2N2M0 | Malignant | 7 | 3 | 10 |  |
| T088a | TMEM156 | A4 | 4 | F | 39 | Breast | Invasive ductal carcinoma | 1 | IIIa | T2N2M0 | Malignant | 7 | 3 | 10 |  |
| T088a | TMEM156 | A5 | 5 | F | 55 | Breast | Invasive ductal carcinoma | 1 | IIb | T2N1M0 | Malignant | 7 | 3 | 10 |  |
| T088a | TMEM156 | A6 | 6 | F | 55 | Breast | Invasive ductal carcinoma | 1 | IIb | T2N1M0 | Malignant | 7 | 3 | 10 |  |
| T088a | TMEM156 | A7 | 7 | F | 39 | Breast | Invasive ductal carcinoma | 1 | IIIa | T2N2M0 | Malignant | 7 | 3 | 10 |  |
| T088a | TMEM156 | A8 | 8 | F | 39 | Breast | Invasive ductal carcinoma | 1 | IIIa | T2N2M0 | Malignant | 7 | 3 | 10 |  |
| T088a | TMEM156 | B1 | 9 | F | 57 | Breast | Invasive ductal carcinoma | 2 | IIa | T2N0M0 | Malignant | 5 | 3 | 8 |  |
| T088a | TMEM156 | B2 | 10 | F | 57 | Breast | Invasive ductal carcinoma | 2 | IIa | T2N0M0 | Malignant | 5 | 3 | 8 |  |
| T088a | TMEM156 | B3 | 11 | F | 47 | Breast | Invasive lobular carcinoma | – | IIa | T2N0M0 | Malignant | 6 | 2 | 8 |  |
| T088a | TMEM156 | B4 | 12 | F | 47 | Breast | Invasive lobular carcinoma | – | IIa | T2N0M0 | Malignant | 6 | 3 | 9 |  |
| T088a | TMEM156 | B5 | 13 | F | 57 | Breast | Invasive ductal carcinoma | 2 | IIa | T2N0M0 | Malignant | 5 | 2 | 7 |  |
| T088a | TMEM156 | B6 | 14 | F | 57 | Breast | Invasive ductal carcinoma | 2 | IIa | T2N0M0 | Malignant | 4 | 2 | 6 |  |
| T088a | TMEM156 | B7 | 15 | F | 47 | Breast | Invasive lobular carcinoma | – | IIa | T2N0M0 | Malignant | 6 | 3 | 9 |  |
| T088a | TMEM156 | B8 | 16 | F | 47 | Breast | Invasive lobular carcinoma | – | IIa | T2N0M0 | Malignant | 6 | 3 | 9 |  |
| T088a | TMEM156 | C1 | 17 | F | 41 | Breast | Cancer adjacent normal breast tissue | – | – | – | NAT | 5 | 2 | 7 |  |
| T088a | TMEM156 | C2 | 18 | F | 41 | Breast | Cancer adjacent normal breast tissue | – | – | – | NAT | 5 | 2 | 7 |  |
| T088a | TMEM156 | C3 | 19 | F | 19 | Breast | Normal breast tissue | – | – | – | Normal | 0 | 0 | 0 |  |
| T088a | TMEM156 | C4 | 20 | F | 19 | Breast | Normal breast tissue | – | – | – | Normal | 0 | 0 | 0 |  |
| T088a | TMEM156 | C5 | 21 | F | 41 | Breast | Cancer adjacent normal breast tissue | – | – | – | NAT | 5 | 2 | 7 |  |
| T088a | TMEM156 | C6 | 22 | F | 41 | Breast | Cancer adjacent normal breast tissue | – | – | – | NAT | 0 | 0 | 0 |  |
| T088a | TMEM156 | C7 | 23 | F | 19 | Breast | Normal breast tissue(fibrofatty tissue and blood vessel) | – | – | – | Normal | 0 | 0 | 0 |  |
| T088a | TMEM156 | C8 | 24 | F | 19 | Breast | Normal breast tissue | – | – | – | Normal | 0 | 0 | 0 |  |

LIVER

| Array | Antibody | Pos | No. | Sex | Age | Organ | Pathology diagnosis | Grade | Stage | TNM | Type † | Proportion Score | Intensity score | Total Score | Comments |
| --- | --- | --- | --- | --- | --- | --- | --- | --- | --- | --- | --- | --- | --- | --- | --- |
| BC03116 | SHISA2 | A1 | 1 | M | 42 | Liver | Hepatocellular carcinoma | 1 | III | T3N0M0 | Malignant | 6 | 2 | 8 |  |
| BC03116 | SHISA2 | A2 | 2 | M | 65 | Liver | Hepatocellular carcinoma | 1 | I | T1N0M0 | Malignant | 5 | 2 | 7 |  |
| BC03116 | SHISA2 | A3 | 3 | M | 50 | Liver | Hepatocellular carcinoma | 1 | III | T3N0M0 | Malignant | 7 | 1 | 8 |  |
| BC03116 | SHISA2 | A4 | 4 | M | 55 | Liver | Hepatocellular carcinoma | 1 | II | T2N0M0 | Malignant | 7 | 2 | 9 |  |
| BC03116 | SHISA2 | A5 | 5 | M | 40 | Liver | Hepatocellular carcinoma | 1 | II | T2N0M0 | Malignant | 7 | 2 | 9 |  |
| BC03116 | SHISA2 | A6 | 6 | M | 52 | Liver | Degenerative hepatocellular carcinoma tissue (sparse) | – | II | T2N0M0 | Malignant | 2 | 1 | 3 |  |
| BC03116 | SHISA2 | A7 | 7 | M | 42 | Liver | Hepatocellular carcinoma | 1 | III | T3N0M0 | Malignant | 6 | 1 | 7 |  |
| BC03116 | SHISA2 | A8 | 8 | M | 40 | Liver | Hepatocellular carcinoma | 1 | II | T2N0M0 | Malignant | 6 | 2 | 8 |  |
| BC03116 | SHISA2 | A9 | 9 | M | 51 | Liver | Hepatocellular carcinoma | 1 | I | T1N0M0 | Malignant | 6 | 2 | 8 |  |
| BC03116 | SHISA2 | A10 | 10 | M | 49 | Liver | Hepatocellular carcinoma | 1 | II | T2N0M0 | Malignant | 3 | 1 | 4 |  |
| BC03116 | SHISA2 | B1 | 11 | M | 38 | Liver | Hepatocellular carcinoma | 1–2 | III | T3N0M0 | Malignant | 7 | 2 | 9 |  |
| BC03116 | SHISA2 | B2 | 12 | F | 46 | Liver | Hepatocellular carcinoma | 1–2 | III | T3N0M0 | Malignant | 7 | 2 | 9 |  |
| BC03116 | SHISA2 | B3 | 13 | M | 48 | Liver | Hepatocellular carcinoma | 2 | III | T3N0M0 | Malignant | 7 | 2 | 9 |  |
| BC03116 | SHISA2 | B4 | 14 | F | 41 | Liver | Hepatocellular carcinoma | 2 | III | T3N0M0 | Malignant | 7 | 1 | 8 |  |
| BC03116 | SHISA2 | B5 | 15 | M | 37 | Liver | Hepatocellular carcinoma | 2 | III | T3N0M0 | Malignant | 7 | 2 | 9 |  |
| BC03116 | SHISA2 | B6 | 16 | M | 59 | Liver | Hepatocellular carcinoma | 2 | III | T3N0M0 | Malignant | 7 | 2 | 9 |  |
| BC03116 | SHISA2 | B7 | 17 | M | 43 | Liver | Hepatocellular carcinoma | 2 | III | T3N0M0 | Malignant | 6 | 1 | 7 |  |
| BC03116 | SHISA2 | B8 | 18 | F | 60 | Liver | Hepatocellular carcinoma | 2 | III | T3N0M0 | Malignant | 7 | 3 | 10 |  |
| BC03116 | SHISA2 | B9 | 19 | M | 50 | Liver | Hepatocellular carcinoma | 2 | III | T2N0M0 | Malignant | 7 | 1 | 8 |  |
| BC03116 | SHISA2 | B10 | 20 | F | 48 | Liver | Hepatocellular carcinoma | 2 | I | T1N0M0 | Malignant | 2 | 1 | 3 |  |
| BC03116 | SHISA2 | C1 | 21 | M | 44 | Liver | Hepatocellular carcinoma | 2 | II | T2N0M0 | Malignant | 7 | 3 | 10 |  |
| BC03116 | SHISA2 | C2 | 22 | M | 73 | Liver | Hepatocellular carcinoma | 2–3 | III | T3N0M0 | Malignant | 7 | 3 | 10 |  |
| BC03116 | SHISA2 | C3 | 23 | M | 41 | Liver | Hepatocellular carcinoma | 2 | III | T3N0M0 | Malignant | 7 | 3 | 10 |  |
| BC03116 | SHISA2 | C4 | 24 | M | 47 | Liver | Hepatocellular carcinoma with necrosis | 2 | II | T2N0M0 | Malignant | 6 | 2 | 8 |  |
| BC03116 | SHISA2 | C5 | 25 | M | 63 | Liver | Hepatocellular carcinoma | 1 | II | T2N0M0 | Malignant | 7 | 2 | 9 |  |
| BC03116 | SHISA2 | C6 | 26 | F | 27 | Liver | Hepatocellular carcinoma | 2 | II | T2N0M0 | Malignant | 6 | 1 | 7 |  |
| BC03116 | SHISA2 | C7 | 27 | F | 63 | Liver | Hepatocellular carcinoma | 2 | III | T3N0M0 | Malignant | 6 | 1 | 7 |  |
| BC03116 | SHISA2 | C8 | 28 | F | 48 | Liver | Hepatocellular carcinoma | 2 | III | T3N0M0 | Malignant | 7 | 2 | 9 |  |
| BC03116 | SHISA2 | C9 | 29 | M | 53 | Liver | Hepatocellular carcinoma | 2 | III | T3N0M0 | Malignant | 5 | 2 | 7 |  |
| BC03116 | SHISA2 | C10 | 30 | M | 42 | Liver | Hepatocellular carcinoma (fibrous tissue and necrosis) | – | II | T2N0M0 | Malignant | 0 | 0 | 0 | No tumor |
| BC03116 | SHISA2 | D1 | 31 | M | 62 | Liver | Hepatocellular carcinoma | 2 | III | T3N0M0 | Malignant | 7 | 2 | 9 |  |
| BC03116 | SHISA2 | D2 | 32 | M | 54 | Liver | Hepatocellular carcinoma | 2 | III | T3N0M0 | Malignant | 7 | 2 | 9 |  |
| BC03116 | SHISA2 | D3 | 33 | M | 53 | Liver | Hepatocellular carcinoma | 2 | III | T3N0M0 | Malignant | 7 | 3 | 10 |  |
| BC03116 | SHISA2 | D4 | 34 | M | 57 | Liver | Hepatocellular carcinoma | 2 | III | T3N0M0 | Malignant | 7 | 2 | 9 |  |
| BC03116 | SHISA2 | D5 | 35 | M | 37 | Liver | Hepatocellular carcinoma | 2 | III | T3N0M0 | Malignant | 5 | 2 | 7 |  |
| BC03116 | SHISA2 | D6 | 36 | M | 70 | Liver | Hepatocellular carcinoma | 2 | II | T2N0M0 | Malignant | 7 | 2 | 9 |  |
| BC03116 | SHISA2 | D7 | 37 | M | 53 | Liver | Hepatocellular carcinoma | 3 | II | T2N0M0 | Malignant | 7 | 3 | 10 |  |
| BC03116 | SHISA2 | D8 | 38 | M | 34 | Liver | Hepatocellular carcinoma | 3 | II | T2N0M0 | Malignant | 7 | 2 | 9 |  |
| BC03116 | SHISA2 | D9 | 39 | M | 66 | Liver | Hepatocellular carcinoma | 3 | I | T1N0M0 | Malignant | 2 | 1 | 3 |  |
| BC03116 | SHISA2 | D10 | 40 | M | 34 | Liver | Hepatocellular carcinoma | 2–3 | III | T3N0M0 | Malignant | 2 | 1 | 3 |  |
| BC03116 | SHISA2 | E1 | 41 | F | 14 | Liver | Normal hepatic tissue | – | – | – | Normal | 7 | 2 | 9 |  |
| BC03116 | SHISA2 | E2 | 42 | F | 2 | Liver | Normal hepatic tissue with focal mild fatty degeneration of hepatocyte | – | – | – | Normal | 7 | 2 | 9 |  |
| BC03116 | SHISA2 | E3 | 43 | M | 56 | Liver | Normal hepatic tissue | – | – | – | Normal | 7 | 3 | 10 |  |
| BC03116 | SHISA2 | E4 | 44 | M | 47 | Liver | Normal hepatic tissue | – | – | – | Normal | 7 | 2 | 9 |  |
| BC03116 | SHISA2 | E5 | 45 | F | 50 | Liver | Normal hepatic tissue with focal mild fatty degeneration of hepatocyte | – | – | – | Normal | 7 | 2 | 9 |  |
| BC03116 | SHISA2 | E6 | 46 | F | 35 | Liver | Normal hepatic tissue with focal mild fatty degeneration of hepatocyte | – | – | – | Normal | 7 | 3 | 10 |  |
| BC03116 | SHISA2 | E7 | 47 | M | 35 | Liver | Normal hepatic tissue | – | – | – | Normal | 7 | 3 | 10 |  |
| BC03116 | SHISA2 | E8 | 48 | M | 35 | Liver | Normal hepatic tissue | – | – | – | Normal | 7 | 3 | 10 |  |
| BC03116 | SHISA2 | E9 | 49 | M | 40 | Liver | Normal hepatic tissue | – | – | – | Normal | 5 | 1 | 6 |  |
| BC03116 | SHISA2 | E10 | 50 | M | 40 | Liver | Normal hepatic tissue | – | – | – | Normal | 5 | 2 | 7 |  |
| BC03116 | SHISA2 | F1 | 51 | M | 38 | Liver | Normal hepatic tissue | – | – | – | Normal | 5 | 2 | 7 |  |
| BC03116 | SHISA2 | F2 | 52 | M | 45 | Liver | Normal hepatic tissue | – | – | – | Normal | 7 | 2 | 9 |  |
| BC03116 | SHISA2 | F3 | 53 | M | 47 | Liver | Normal hepatic tissue | – | – | – | Normal | 7 | 2 | 9 |  |
| BC03116 | SHISA2 | F4 | 54 | M | 16 | Liver | Normal hepatic tissue | – | – | – | Normal | 7 | 2 | 9 |  |
| BC03116 | SHISA2 | F5 | 55 | F | 18 | Liver | Normal hepatic tissue | – | – | – | Normal | 7 | 3 | 10 |  |
| BC03116 | SHISA2 | F6 | 56 | F | 21 | Liver | Normal hepatic tissue | – | – | – | Normal | 7 | 2 | 9 |  |
| BC03116 | SHISA2 | F7 | 57 | M | 43 | Liver | Normal hepatic tissue | – | – | – | Normal | 7 | 2 | 9 |  |
| BC03116 | SHISA2 | F8 | 58 | M | 35 | Liver | Cancer adjacent normal hepatic tissue | – | – | – | NAT | 6 | 2 | 8 |  |
| BC03116 | SHISA2 | F9 | 59 | M | 56 | Liver | Cancer adjacent normal hepatic tissue | – | – | – | NAT | 7 | 2 | 9 |  |
| BC03116 | SHISA2 | F10 | 60 | F | 61 | Liver | Cancer adjacent normal hepatic tissue | – | – | – | NAT | 6 | 2 | 8 |  |
| BC03116 | SHISA2 | G1 | 61 | F | 57 | Liver | Cancer adjacent normal hepatic tissue ( with lymphocyte infiltrating) | – | – | – | NAT | 4 | 1 | 5 |  |
| BC03116 | SHISA2 | G2 | 62 | M | 60 | Liver | Cancer adjacent normal hepatic tissue | – | – | – | NAT | 4 | 1 | 5 |  |
| BC03116 | SHISA2 | G3 | 63 | F | 65 | Liver | Cancer adjacent normal hepatic tissue | – | – | – | NAT | 5 | 2 | 7 |  |
| BC03116 | SHISA2 | G4 | 64 | M | 32 | Liver | Cancer adjacent normal hepatic tissue | – | – | – | NAT | 4 | 1 | 5 |  |
| BC03116 | SHISA2 | G5 | 65 | M | 31 | Liver | Cancer adjacent normal hepatic tissue | – | – | – | NAT | 3 | 1 | 4 |  |
| BC03116 | SHISA2 | G6 | 66 | M | 31 | Liver | Cancer adjacent normal hepatic tissue | – | – | – | NAT | 4 | 2 | 6 |  |
| BC03116 | SHISA2 | G7 | 67 | M | 27 | Liver | Cancer adjacent normal hepatic tissue (sparse) and hyperplastic fibrous tissue and bile duct | – | – | – | NAT | 3 | 1 | 4 | Scant liver tissue |
| BC03116 | SHISA2 | G8 | 68 | F | 35 | Liver | Cancer adjacent normal hepatic tissue | – | – | – | NAT | 4 | 2 | 6 |  |
| BC03116 | SHISA2 | G9 | 69 | F | 25 | Liver | Cancer adjacent normal hepatic tissue | – | – | – | NAT | 3 | 1 | 4 |  |
| BC03116 | SHISA2 | G10 | 70 | F | 23 | Liver | Cancer adjacent normal hepatic tissue (with lymphocyte infiltrating) | – | – | – | NAT | 2 | 1 | 3 |  |
| T031 | TMEM156 | A1 | 1 | M | 49 | Liver | Hepatocellular carcinoma | 2 | II | T2N0M0 | Malignant | 6 | 3 | 9 |  |
| T031 | TMEM156 | A2 | 2 | M | 49 | Liver | Hepatocellular carcinoma | 2 | II | T2N0M0 | Malignant | 7 | 3 | 10 |  |
| T031 | TMEM156 | A3 | 3 | M | 57 | Liver | Hepatocholangiocarcinoma | 2 | III | T3N0M0 | Malignant | 7 | 3 | 10 |  |
| T031 | TMEM156 | A4 | 4 | M | 57 | Liver | Hepatocholangiocarcinoma | 2 | III | T3N0M0 | Malignant | 7 | 3 | 10 |  |
| T031 | TMEM156 | A5 | 5 | M | 49 | Liver | Hepatocellular carcinoma | 2 | II | T2N0M0 | Malignant | 7 | 3 | 10 |  |
| T031 | TMEM156 | A6 | 6 | M | 49 | Liver | Hepatocellular carcinoma | 2 | II | T2N0M0 | Malignant | 7 | 3 | 10 |  |
| T031 | TMEM156 | A7 | 7 | M | 57 | Liver | Hepatocholangiocarcinoma | 2 | III | T3N0M0 | Malignant | na | na | na | No Hepatocellular Carcinoma, Only Adenocarcinoma. |
| T031 | TMEM156 | A8 | 8 | M | 57 | Liver | Hepatocholangiocarcinoma | 2 | III | T3N0M0 | Malignant | 6 | 3 | 9 |  |
| T031 | TMEM156 | B1 | 9 | M | 61 | Liver | Hepatocellular carcinoma | 1–2 | II | T2N0M0 | Malignant | 5 | 3 | 8 |  |
| T031 | TMEM156 | B2 | 10 | M | 61 | Liver | Hepatocellular carcinoma | 1–2 | II | T2N0M0 | Malignant | 5 | 3 | 8 |  |
| T031 | TMEM156 | B3 | 11 | M | 62 | Liver | Hepatocellular carcinoma | 3 | IIIA | T3N0M0 | Malignant | 0 | 0 | 0 |  |
| T031 | TMEM156 | B4 | 12 | M | 62 | Liver | Hepatocellular carcinoma | 3 | IIIA | T3N0M0 | Malignant | 0 | 0 | 0 |  |
| T031 | TMEM156 | B5 | 13 | M | 61 | Liver | Hepatocellular carcinoma | 1–2 | II | T2N0M0 | Malignant | 3 | 3 | 6 |  |
| T031 | TMEM156 | B6 | 14 | M | 61 | Liver | Hepatocellular carcinoma | 1–2 | II | T2N0M0 | Malignant | 7 | 3 | 10 |  |
| T031 | TMEM156 | B7 | 15 | M | 62 | Liver | Hepatocellular carcinoma | 3 | IIIA | T3N0M0 | Malignant | 0 | 0 | 0 |  |
| T031 | TMEM156 | B8 | 16 | M | 62 | Liver | Hepatocellular carcinoma | 3 | IIIA | T3N0M0 | Malignant | 0 | 0 | 0 |  |
| T031 | TMEM156 | C1 | 17 | M | 43 | Liver | Normal hepatic tissue | – | – | – | Normal | 0 | 0 | 0 |  |
| T031 | TMEM156 | C2 | 18 | M | 43 | Liver | Normal hepatic tissue | – | – | – | Normal | 0 | 0 | 0 |  |
| T031 | TMEM156 | C3 | 19 | F | 50 | Liver | Normal hepatic tissue | – | – | – | Normal | 0 | 0 | 0 |  |
| T031 | TMEM156 | C4 | 20 | F | 50 | Liver | Normal hepatic tissue | – | – | – | Normal | 0 | 0 | 0 |  |
| T031 | TMEM156 | C5 | 21 | M | 43 | Liver | Normal hepatic tissue | – | – | – | Normal | 0 | 0 | 0 |  |
| T031 | TMEM156 | C6 | 22 | M | 43 | Liver | Normal hepatic tissue | – | – | – | Normal | 0 | 0 | 0 |  |
| T031 | TMEM156 | C7 | 23 | F | 50 | Liver | Normal hepatic tissue | – | – | – | Normal | 0 | 0 | 0 |  |
| T031 | TMEM156 | C8 | 24 | F | 50 | Liver | Normal hepatic tissue | – | – | – | Normal | 0 | 0 | 0 |  |
| T031 | TMEM156 | – | – | M | 42 | Adrenal gland | Pheochromocytoma (tissue marker) | – |  |  | Malignant | 3 | 3 | 6 |  |

PROSTATE

| Array | Antibody | Pos | No. | Sex | Age | Organ | Pathology diagnosis | Grade | Stage | Gleason Grade | Gleason Score | TNM | Type † | Proportion Score | Intensity Score | Total Score | Comments |
| --- | --- | --- | --- | --- | --- | --- | --- | --- | --- | --- | --- | --- | --- | --- | --- | --- | --- |
| PR633 | C11orf68 | A1 | 1 | M | 71 | Prostate | Adenocarcinoma | 1 | II | 2 | 1+2 | T2N0M0 | Malignant | 0 | 0 | 0 |  |
| PR633 | C11orf68 | A2 | 2 | M | 60 | Prostate | Adenocarcinoma (sparse) | 1 | III | 2 | 2+2 | T3aN0M0 | Malignant | 0 | 0 | 0 |  |
| PR633 | C11orf68 | A3 | 3 | M | 66 | Prostate | Adenocarcinoma | 1 | IV | 2 | 1+2 | T3N1M1 | Malignant | 0 | 0 | 0 |  |
| PR633 | C11orf68 | A4 | 4 | M | 71 | Prostate | Adenocarcinoma | 1 | I | 2 | 2+2 | T1N0M0 | Malignant | 0 | 0 | 0 |  |
| PR633 | C11orf68 | A5 | 5 | M | 71 | Prostate | Adenocarcinoma | 1 | II | 2 | 1+2 | T2N0M0 | Malignant | 0 | 0 | 0 |  |
| PR633 | C11orf68 | A6 | 6 | M | 76 | Prostate | Adenocarcinoma | 1 | II | 2 | 2+2 | T2aN0M0 | Malignant | 0 | 0 | 0 |  |
| PR633 | C11orf68 | A7 | 7 | M | 72 | Prostate | Adenocarcinoma | 1 | II | 2 | 1+2 | T2N0M0 | Malignant | 0 | 0 | 0 |  |
| PR633 | C11orf68 | A8 | 8 | M | 73 | Prostate | Adenocarcinoma | 1 | III | 2 | 2+2 | T3N0M0 | Malignant | 0 | 0 | 0 |  |
| PR633 | C11orf68 | A9 | 9 | M | 71 | Prostate | Adenocarcinoma | 2 | II | 3 | 2+3 | T2N0M0 | Malignant | 0 | 0 | 0 |  |
| PR633 | C11orf68 | B1 | 10 | M | 74 | Prostate | Adenocarcinoma | 1 | IV | 2 | 2+2 | T4N1M1 | Malignant | 0 | 0 | 0 |  |
| PR633 | C11orf68 | B2 | 11 | M | 75 | Prostate | Adenocarcinoma | 2 | IV | 3 | 2+4 | T4N1M1 | Malignant | 5 | 1 | 6 |  |
| PR633 | C11orf68 | B3 | 12 | M | 69 | Prostate | Adenocarcinoma (sparse) | 1 | II | 2 | 1+2 | T2N0M0 | Malignant | 0 | 0 | 0 |  |
| PR633 | C11orf68 | B4 | 13 | M | 78 | Prostate | Adenocarcinoma | 2 | III | 3 | 2+4 | T3N2M1 | Malignant | 6 | 1 | 7 |  |
| PR633 | C11orf68 | B5 | 14 | M | 73 | Prostate | Adenocarcinoma | 2 | II | 3 | 3+3 | T2N0M0 | Malignant | 7 | 1 | 8 |  |
| PR633 | C11orf68 | B6 | 15 | M | 65 | Prostate | Adenocarcinoma | 2 | II | 3 | 3+3 | T2N0M0 | Malignant | 0 | 0 | 0 |  |
| PR633 | C11orf68 | B7 | 16 | M | 73 | Prostate | Adenocarcinoma (cataplasia tissue) | – | II | – | – | T2N0M0 | Malignant | 0 | 0 | 0 |  |
| PR633 | C11orf68 | B8 | 17 | M | 70 | Prostate | Adenocarcinoma | 2 | IV | 3 | 3+3 | T2N1M1c | Malignant | 0 | 0 | 0 |  |
| PR633 | C11orf68 | B9 | 18 | M | 58 | Prostate | Adenocarcinoma | 2 | II | 3 | 3+3 | T2N0M0 | Malignant | 0 | 0 | 0 |  |
| PR633 | C11orf68 | C1 | 19 | M | 64 | Prostate | Adenocarcinoma | 2 | II | 3 | 3+3 | T2aN0M0 | Malignant | 0 | 0 | 0 |  |
| PR633 | C11orf68 | C2 | 20 | M | 62 | Prostate | Adenocarcinoma | 2 | II | 3 | 3+3 | T2N0M0 | Malignant | 2 | 1 | 3 |  |
| PR633 | C11orf68 | C3 | 21 | M | 60 | Prostate | Adenocarcinoma | 2 | III | 3 | 2+4 | T3N1M0 | Malignant | 2 | 1 | 3 |  |
| PR633 | C11orf68 | C4 | 22 | M | 65 | Prostate | Adenocarcinoma | 2 | II | 3 | 3+3 | T2N0M0 | Malignant | 0 | 0 | 0 |  |
| PR633 | C11orf68 | C5 | 23 | M | 82 | Prostate | Adenocarcinoma | 1 | IV | 2 | 2+2 | T3N2M1c | Malignant | 0 | 0 | 0 |  |
| PR633 | C11orf68 | C6 | 24 | M | 64 | Prostate | Adenocarcinoma | 2 | II | 3 | 2+4 | T2N0M0 | Malignant | 0 | 0 | 0 |  |
| PR633 | C11orf68 | C7 | 25 | M | 65 | Prostate | Adenocarcinoma | 2 | IV | 3 | 3+3 | T2N1M1 | Malignant | 0 | 0 | 0 |  |
| PR633 | C11orf68 | C8 | 26 | M | 73 | Prostate | Adenocarcinoma | 2 | IV | 3 | 3+3 | T3N1M1b | Malignant | 0 | 0 | 0 |  |
| PR633 | C11orf68 | C9 | 27 | M | 70 | Prostate | Adenocarcinoma | 3 | II | 4 | 4+4 | T2N0M0 | Malignant | 0 | 0 | 0 |  |
| PR633 | C11orf68 | D1 | 28 | M | 72 | Prostate | Adenocarcinoma | 3 | III | 4 | 4+5 | T3N0M0 | Malignant | 0 | 0 | 0 |  |
| PR633 | C11orf68 | D2 | 29 | M | 26 | Prostate | Adenocarcinoma | 3 | II | 4 | 4+4 | T2N0M0 | Malignant | 0 | 0 | 0 |  |
| PR633 | C11orf68 | D3 | 30 | M | 62 | Prostate | Adenocarcinoma | 2–3 | IV | 3–4 | 4+3 | T3N1M1b | Malignant | 0 | 0 | 0 |  |
| PR633 | C11orf68 | D4 | 31 | M | 64 | Prostate | Adenocarcinoma | 2 | II | 3 | 3+3 | T2N0M0 | Malignant | 2 | 1 | 3 |  |
| PR633 | C11orf68 | D5 | 32 | M | 64 | Prostate | Adenocarcinoma | 2 | IV | 3 | 2+4 | T3N0M1b | Malignant | 4 | 1 | 5 |  |
| PR633 | C11orf68 | D6 | 33 | M | 69 | Prostate | Adenocarcinoma | 2 | III | 3 | 2+4 | T3bN0M0 | Malignant | 0 | 0 | 0 |  |
| PR633 | C11orf68 | D7 | 34 | M | 51 | Prostate | Adenocarcinoma | 3 | II | 4 | 4+5 | T2N0M0 | Malignant | 3 | 1 | 4 |  |
| PR633 | C11orf68 | D8 | 35 | M | 73 | Prostate | Adenocarcinoma | 3 | III | 4 | 4+5 | T3N1M1 | Malignant | 6 | 1 | 7 |  |
| PR633 | C11orf68 | D9 | 36 | M | 78 | Prostate | Adenocarcinoma | 2 | III | 3 | 3+4 | T4N0M0 | Malignant | 0 | 0 | 0 |  |
| PR633 | C11orf68 | E1 | 37 | M | 20 | Prostate | Adenocarcinoma | 2 | III | 3 | 3+4 | T3N0M0 | Malignant | 0 | 0 | 0 |  |
| PR633 | C11orf68 | E2 | 38 | M | 66 | Prostate | Adenocarcinoma | 2–3 | III | 4 | 4+3 | T3aN0M0 | Malignant | 3 | 1 | 4 |  |
| PR633 | C11orf68 | E3 | 39 | M | 80 | Prostate | Adenocarcinoma | 2 | IV | 3 | 3+3 | T4N1M1c | Malignant | 0 | 0 | 0 |  |
| PR633 | C11orf68 | E4 | 40 | M | 70 | Prostate | Adenocarcinoma (smooth muscle) | – | III | – | – | T3N0M0 | Malignant | 0 | 0 | 0 |  |
| PR633 | C11orf68 | E5 | 41 | M | 81 | Prostate | Adenocarcinoma | 3 | III | 5 | 4+5 | T3aN0M0 | Malignant | 0 | 0 | 0 |  |
| PR633 | C11orf68 | E6 | 42 | M | 61 | Prostate | Adenocarcinoma | 3 | III | 5 | 4+5 | T3N1M0 | Malignant | 0 | 0 | 0 |  |
| PR633 | C11orf68 | E7 | 43 | M | 40 | Prostate | Adenocarcinoma (hyperplasia) | – | II | – | – | T2N0M0 | Malignant | 0 | 0 | 0 |  |
| PR633 | C11orf68 | E8 | 44 | M | 76 | Prostate | Adenocarcinoma (hyperplasia) | – | III | – | – | T3N0M0 | Malignant | 0 | 0 | 0 |  |
| PR633 | C11orf68 | E9 | 45 | M | 64 | Prostate | Adenocarcinoma | 3 | II | 4 | 4+5 | T2N0M0 | Malignant | 1 | 1 | 2 |  |
| PR633 | C11orf68 | F1 | 46 | M | 69 | Prostate | Adenocarcinoma | 2 | II | 3 | 3+5 | T2N0M0 | Malignant | 0 | 0 | 0 |  |
| PR633 | C11orf68 | F2 | 47 | M | 62 | Prostate | Adenocarcinoma | 3 | II | 4 | 4+4 | T2N0M0 | Malignant | 0 | 0 | 0 |  |
| PR633 | C11orf68 | F3 | 48 | M | 82 | Prostate | Adenocarcinoma | 3 | II | 4 | 4+4 | T2N0M0 | Malignant | 0 | 0 | 0 |  |
| PR633 | C11orf68 | F4 | 49 | M | 75 | Prostate | Adenocarcinoma | 3 | II | 5 | 5+4 | T2N0M0 | Malignant | 0 | 0 | 0 |  |
| PR633 | C11orf68 | F5 | 50 | M | 73 | Prostate | Adenocarcinoma | 3 | III | 5 | 5+4 | T4N0M0 | Malignant | 0 | 0 | 0 |  |
| PR633 | C11orf68 | F6 | 51 | M | 67 | Prostate | Adenocarcinoma | 3 | II | 5 | 5+4 | T2N0M0 | Malignant | 0 | 0 | 0 |  |
| PR633 | C11orf68 | F7 | 52 | M | 75 | Prostate | Adenocarcinoma | 3 | III | 5 | 5+5 | T3N1M0 | Malignant | 0 | 0 | 0 |  |
| PR633 | C11orf68 | F8 | 53 | M | 60 | Prostate | Adenocarcinoma | 3 | II | 5 | 5+5 | T2N0M0 | Malignant | 0 | 0 | 0 |  |
| PR633 | C11orf68 | F9 | 54 | M | 60 | Prostate | Adenocarcinoma | 3 | IV | 5 | 5+4 | T3N1M1b | Malignant | 0 | 0 | 0 |  |
| PR633 | C11orf68 | G1 | 55 | M | 63 | Prostate | Adenocarcinoma | 3 | II | 5 | 5+4 | T2N0M0 | Malignant | 0 | 0 | 0 |  |
| PR633 | C11orf68 | G2 | 56 | M | 56 | Prostate | Adenocarcinoma | 3 | II | 5 | 5+5 | T2N0M0 | Malignant | 0 | 0 | 0 |  |
| PR633 | C11orf68 | G3 | 57 | M | 76 | Prostate | Adenocarcinoma | 3 | II | 4 | 4+4 | T2aN0M0 | Malignant | 0 | 0 | 0 |  |
| PR633 | C11orf68 | G4 | 58 | M | 77 | Prostate | Adenocarcinoma | 3 | II | 5 | 5+4 | T2N0M0 | Malignant | 3 | 1 | 4 |  |
| PR633 | C11orf68 | G5 | 59 | M | 75 | Prostate | Adenocarcinoma | 3 | III | 5 | 5+5 | T3N0M0 | Malignant | 0 | 0 | 0 |  |
| PR633 | C11orf68 | G6 | 60 | M | 66 | Prostate | Adenocarcinoma | 3 | II | 5 | 5+5 | T2N0M0 | Malignant | 0 | 0 | 0 |  |
| PR633 | C11orf68 | G7 | 61 | M | 36 | Prostate | Normal prostate tissue | – |  | – | – |  | Normal | 0 | 0 | 0 |  |
| PR633 | C11orf68 | G8 | 62 | M | 37 | Prostate | Normal prostate tissue | – | – | – | – | – | Normal | 0 | 0 | 0 |  |
| PR633 | C11orf68 | G9 | 63 | M | 31 | Prostate | Normal prostate tissue | – | – | – | – | – | Normal | 0 | 0 | 0 |  |
| PR633 | C11orf68 | – | – | M | 42 | Adrenal gland | Pheochromocytoma (tissue marker) | – |  |  |  |  | Malignant | 6 | 1 | 7 |  |
| T195b | C11orf68 | A1 | 1 | M | 64 | Prostate | Adenocarcinoma | 1 | I | 1 | 1+2 | T1N0M0 | Malignant | 0 | 0 | 0 |  |
| T195b | C11orf68 | A2 | 2 | M | 73 | Prostate | Adenocarcinoma | 2 | II | 3 | 3+3 | T2N0M0 | Malignant | 0 | 0 | 0 |  |
| T195b | C11orf68 | A3 | 3 | M | 73 | Prostate | Adenocarcinoma | 2–3 | IV | 4 | 4+4 | T3N0M1 | Malignant | 6 | 2 | 8 |  |
| T195b | C11orf68 | A4 | 4 | M | 61 | Prostate | Adenocarcinoma | 1 | IV | 2 | 3+2 | T3N1M0 | Malignant | 2 | 1 | 3 |  |
| T195b | C11orf68 | A5 | 5 | M | 64 | Prostate | Adenocarcinoma | 1 | I | 1 | 1+2 | T1N0M0 | Malignant | 0 | 0 | 0 |  |
| T195b | C11orf68 | A6 | 6 | M | 73 | Prostate | Adenocarcinoma | 2 | II | 3 | 3+3 | T2N0M0 | Malignant | 0 | 0 | 0 |  |
| T195b | C11orf68 | A7 | 7 | M | 73 | Prostate | Adenocarcinoma | 2–3 | IV | 4 | 4+4 | T3N0M1 | Malignant | 3 | 1 | 4 |  |
| T195b | C11orf68 | A8 | 8 | M | 61 | Prostate | Adenocarcinoma | 1 | IV | 2 | 3+2 | T3N1M0 | Malignant | 2 | 1 | 3 |  |
| T195b | C11orf68 | B1 | 9 | M | 70 | Prostate | Adenocarcinoma | 2 | III | 3 | 3+4 | T3N0M0 | Malignant | 0 | 0 | 0 |  |
| T195b | C11orf68 | B2 | 10 | M | 66 | Prostate | Adenocarcinoma | 2–3 | III | 4 | 3+4 | T3aN0M0 | Malignant | 5 | 1 | 6 |  |
| T195b | C11orf68 | B3 | 11 | M | 65 | Prostate | Adenocarcinoma | 1–2 | II | 2–3 | 2+3 | T2N0M0 | Malignant | 2 | 1 | 3 |  |
| T195b | C11orf68 | B4 | 12 | M | 66 | Prostate | Adenocarcinoma | 3 | II | 5 | 5+5 | T2N0M0 | Malignant | 5 | 1 | 6 |  |
| T195b | C11orf68 | B5 | 13 | M | 70 | Prostate | Adenocarcinoma | 2 | III | 3 | 3+4 | T3N0M0 | Malignant | 0 | 0 | 0 |  |
| T195b | C11orf68 | B6 | 14 | M | 66 | Prostate | Adenocarcinoma | 2–3 | III | 4 | 3+4 | T3aN0M0 | Malignant | 4 | 1 | 5 |  |
| T195b | C11orf68 | B7 | 15 | M | 65 | Prostate | Adenocarcinoma | 1–2 | II | 2–3 | 2+3 | T2N0M0 | Malignant | 2 | 1 | 3 |  |
| T195b | C11orf68 | B8 | 16 | M | 66 | Prostate | Adenocarcinoma | 3 | II | 5 | 5+5 | T2N0M0 | Malignant | 3 | 1 | 4 |  |
| T195b | C11orf68 | C1 | 17 | M | 62 | Prostate | Adenocarcinoma | 3 | II | 5 | 5+4 | T2N0M0 | Malignant | 0 | 0 | 0 |  |
| T195b | C11orf68 | C2 | 18 | M | 69 | Prostate | Low grade malignant leiomyosarcoma | – | Ia | – | – | T1N0M0 G1 | Malignant | 0 | 0 | 0 |  |
| T195b | C11orf68 | C3 | 19 | M | 33 | Prostate | Normal prostate tissue | – | – | – | – | – | Normal | 0 | 0 | 0 |  |
| T195b | C11orf68 | C4 | 20 | M | 43 | Prostate | Normal prostate tissue | – | – | – | – | – | Normal | 0 | 0 | 0 |  |
| T195b | C11orf68 | C5 | 21 | M | 62 | Prostate | Adenocarcinoma | 2 | II | 3 | 3+4 | T2N0M0 | Malignant | 0 | 0 | 0 |  |
| T195b | C11orf68 | C6 | 22 | M | 69 | Prostate | Low grade malignant leiomyosarcoma | – | Ia | – | – | T1N0M0 G1 | Malignant | 0 | 0 | 0 |  |
| T195b | C11orf68 | C7 | 23 | M | 33 | Prostate | Normal prostate tissue | – | – | – | – | – | Normal | 0 | 0 | 0 |  |
| T195b | C11orf68 | C8 | 24 | M | 43 | Prostate | Normal prostate tissue | – | – | – | – | – | Normal | 0 | 0 | 0 |  |
| T195b | C11orf68 | – | – | M | 58 | Skin | Malignant melanoma (tissue marker) | – |  |  |  |  | Malignant | 7 | 1 | 8 |  |
| PR633 | SHISA2 | – | – | M | 42 | Adrenal gland | Pheochromocytoma (tissue marker) | – |  |  |  |  | Malignant | 6 | 1 | 7 |  |
| PR633 | SHISA2 | A1 | 1 | M | 71 | Prostate | Adenocarcinoma | 1 | II | 2 | 1+2 | T2N0M0 | Malignant | 6 | 1 | 7 |  |
| PR633 | SHISA2 | A2 | 2 | M | 60 | Prostate | Adenocarcinoma (sparse) | 1 | III | 2 | 2+2 | T3aN0M0 | Malignant | 6 | 2 | 8 | Only few tumor cells |
| PR633 | SHISA2 | A3 | 3 | M | 66 | Prostate | Adenocarcinoma | 1 | IV | 2 | 1+2 | T3N1M1 | Malignant | 5 | 1 | 6 |  |
| PR633 | SHISA2 | A4 | 4 | M | 71 | Prostate | Adenocarcinoma | 1 | I | 2 | 2+2 | T1N0M0 | Malignant | 2 | 1 | 3 |  |
| PR633 | SHISA2 | A5 | 5 | M | 71 | Prostate | Adenocarcinoma | 1 | II | 2 | 1+2 | T2N0M0 | Malignant | 6 | 1 | 7 |  |
| PR633 | SHISA2 | A6 | 6 | M | 76 | Prostate | Adenocarcinoma | 1 | II | 2 | 2+2 | T2aN0M0 | Malignant | 5 | 1 | 6 |  |
| PR633 | SHISA2 | A7 | 7 | M | 72 | Prostate | Adenocarcinoma | 1 | II | 2 | 1+2 | T2N0M0 | Malignant | 1 | 1 | 2 |  |
| PR633 | SHISA2 | A8 | 8 | M | 73 | Prostate | Adenocarcinoma | 1 | III | 2 | 2+2 | T3N0M0 | Malignant | 3 | 1 | 4 |  |
| PR633 | SHISA2 | A9 | 9 | M | 71 | Prostate | Adenocarcinoma | 2 | II | 3 | 2+3 | T2N0M0 | Malignant | 2 | 1 | 3 |  |
| PR633 | SHISA2 | B1 | 10 | M | 74 | Prostate | Adenocarcinoma | 1 | IV | 2 | 2+2 | T4N1M1 | Malignant | 5 | 1 | 6 |  |
| PR633 | SHISA2 | B2 | 11 | M | 75 | Prostate | Adenocarcinoma | 2 | IV | 3 | 2+4 | T4N1M1 | Malignant | 6 | 2 | 8 |  |
| PR633 | SHISA2 | B3 | 12 | M | 69 | Prostate | Adenocarcinoma (sparse) | 1 | II | 2 | 1+2 | T2N0M0 | Malignant | 1 | 1 | 2 |  |
| PR633 | SHISA2 | B4 | 13 | M | 78 | Prostate | Adenocarcinoma | 2 | III | 3 | 2+4 | T3N2M1 | Malignant | 7 | 2 | 9 |  |
| PR633 | SHISA2 | B5 | 14 | M | 73 | Prostate | Adenocarcinoma | 2 | II | 3 | 3+3 | T2N0M0 | Malignant | 6 | 2 | 8 |  |
| PR633 | SHISA2 | B6 | 15 | M | 65 | Prostate | Adenocarcinoma | 2 | II | 3 | 3+3 | T2N0M0 | Malignant | 4 | 1 | 5 |  |
| PR633 | SHISA2 | B7 | 16 | M | 73 | Prostate | Adenocarcinoma (cataplasia tissue) | – | II | – | – | T2N0M0 | Malignant | 0 | 0 | 0 |  |
| PR633 | SHISA2 | B8 | 17 | M | 70 | Prostate | Adenocarcinoma | 2 | IV | 3 | 3+3 | T2N1M1c | Malignant | 0 | 0 | 0 |  |
| PR633 | SHISA2 | B9 | 18 | M | 58 | Prostate | Adenocarcinoma | 2 | II | 3 | 3+3 | T2N0M0 | Malignant | 2 | 1 | 3 |  |
| PR633 | SHISA2 | C1 | 19 | M | 64 | Prostate | Adenocarcinoma | 2 | II | 3 | 3+3 | T2aN0M0 | Malignant | 1 | 1 | 2 |  |
| PR633 | SHISA2 | C2 | 20 | M | 62 | Prostate | Adenocarcinoma | 2 | II | 3 | 3+3 | T2N0M0 | Malignant | 7 | 2 | 9 |  |
| PR633 | SHISA2 | C3 | 21 | M | 60 | Prostate | Adenocarcinoma | 2 | III | 3 | 2+4 | T3N1M0 | Malignant | 7 | 2 | 9 |  |
| PR633 | SHISA2 | C4 | 22 | M | 65 | Prostate | Adenocarcinoma | 2 | II | 3 | 3+3 | T2N0M0 | Malignant | 4 | 1 | 5 |  |
| PR633 | SHISA2 | C5 | 23 | M | 82 | Prostate | Adenocarcinoma | 1 | IV | 2 | 2+2 | T3N2M1c | Malignant | 2 | 1 | 3 |  |
| PR633 | SHISA2 | C6 | 24 | M | 64 | Prostate | Adenocarcinoma | 2 | II | 3 | 2+4 | T2N0M0 | Malignant | 6 | 1 | 7 |  |
| PR633 | SHISA2 | C7 | 25 | M | 65 | Prostate | Adenocarcinoma | 2 | IV | 3 | 3+3 | T2N1M1 | Malignant | 5 | 1 | 6 |  |
| PR633 | SHISA2 | C8 | 26 | M | 73 | Prostate | Adenocarcinoma | 2 | IV | 3 | 3+3 | T3N1M1b | Malignant | 0 | 0 | 0 |  |
| PR633 | SHISA2 | C9 | 27 | M | 70 | Prostate | Adenocarcinoma | 3 | II | 4 | 4+4 | T2N0M0 | Malignant | 0 | 0 | 0 |  |
| PR633 | SHISA2 | D1 | 28 | M | 72 | Prostate | Adenocarcinoma | 3 | III | 4 | 4+5 | T3N0M0 | Malignant | 5 | 1 | 6 |  |
| PR633 | SHISA2 | D2 | 29 | M | 26 | Prostate | Adenocarcinoma | 3 | II | 4 | 4+4 | T2N0M0 | Malignant | 5 | 1 | 6 |  |
| PR633 | SHISA2 | D3 | 30 | M | 62 | Prostate | Adenocarcinoma | 2–3 | IV | 3–4 | 4+3 | T3N1M1b | Malignant | 6 | 2 | 8 |  |
| PR633 | SHISA2 | D4 | 31 | M | 64 | Prostate | Adenocarcinoma | 2 | II | 3 | 3+3 | T2N0M0 | Malignant | 4 | 1 | 5 |  |
| PR633 | SHISA2 | D5 | 32 | M | 64 | Prostate | Adenocarcinoma | 2 | IV | 3 | 2+4 | T3N0M1b | Malignant | 5 | 1 | 6 |  |
| PR633 | SHISA2 | D6 | 33 | M | 69 | Prostate | Adenocarcinoma | 2 | III | 3 | 2+4 | T3bN0M0 | Malignant | 0 | 0 | 0 |  |
| PR633 | SHISA2 | D7 | 34 | M | 51 | Prostate | Adenocarcinoma | 3 | II | 4 | 4+5 | T2N0M0 | Malignant | 3 | 1 | 4 |  |
| PR633 | SHISA2 | D8 | 35 | M | 73 | Prostate | Adenocarcinoma | 3 | III | 4 | 4+5 | T3N1M1 | Malignant | 0 | 0 | 0 |  |
| PR633 | SHISA2 | D9 | 36 | M | 78 | Prostate | Adenocarcinoma | 2 | III | 3 | 3+4 | T4N0M0 | Malignant | 1 | 1 | 2 |  |
| PR633 | SHISA2 | E1 | 37 | M | 20 | Prostate | Adenocarcinoma | 2 | III | 3 | 3+4 | T3N0M0 | Malignant | 0 | 0 | 0 |  |
| PR633 | SHISA2 | E2 | 38 | M | 66 | Prostate | Adenocarcinoma | 2–3 | III | 4 | 4+3 | T3aN0M0 | Malignant | 7 | 2 | 9 |  |
| PR633 | SHISA2 | E3 | 39 | M | 80 | Prostate | Adenocarcinoma | 2 | IV | 3 | 3+3 | T4N1M1c | Malignant | 2 | 1 | 3 |  |
| PR633 | SHISA2 | E4 | 40 | M | 70 | Prostate | Adenocarcinoma (smooth muscle) | – | III | – | – | T3N0M0 | Malignant | 0 | 0 | 0 | Only few tumor cells |
| PR633 | SHISA2 | E5 | 41 | M | 81 | Prostate | Adenocarcinoma | 3 | III | 5 | 4+5 | T3aN0M0 | Malignant | 0 | 0 | 0 |  |
| PR633 | SHISA2 | E6 | 42 | M | 61 | Prostate | Adenocarcinoma | 3 | III | 5 | 4+5 | T3N1M0 | Malignant | 2 | 1 | 3 |  |
| PR633 | SHISA2 | E7 | 43 | M | 40 | Prostate | Adenocarcinoma (hyperplasia) | – | II | – | – | T2N0M0 | Malignant | na | na | na | No tumor |
| PR633 | SHISA2 | E8 | 44 | M | 76 | Prostate | Adenocarcinoma (hyperplasia) | – | III | – | – | T3N0M0 | Malignant | na | na | na | No tumor |
| PR633 | SHISA2 | E9 | 45 | M | 64 | Prostate | Adenocarcinoma | 3 | II | 4 | 4+5 | T2N0M0 | Malignant | 4 | 1 | 5 |  |
| PR633 | SHISA2 | F1 | 46 | M | 69 | Prostate | Adenocarcinoma | 2 | II | 3 | 3+5 | T2N0M0 | Malignant | 2 | 1 | 3 |  |
| PR633 | SHISA2 | F2 | 47 | M | 62 | Prostate | Adenocarcinoma | 3 | II | 4 | 4+4 | T2N0M0 | Malignant | 0 | 0 | 0 |  |
| PR633 | SHISA2 | F3 | 48 | M | 82 | Prostate | Adenocarcinoma | 3 | II | 4 | 4+4 | T2N0M0 | Malignant | 6 | 2 | 8 |  |
| PR633 | SHISA2 | F4 | 49 | M | 75 | Prostate | Adenocarcinoma | 3 | II | 5 | 5+4 | T2N0M0 | Malignant | 2 | 1 | 3 |  |
| PR633 | SHISA2 | F5 | 50 | M | 73 | Prostate | Adenocarcinoma | 3 | III | 5 | 5+4 | T4N0M0 | Malignant | 6 | 1 | 7 |  |
| PR633 | SHISA2 | F6 | 51 | M | 67 | Prostate | Adenocarcinoma | 3 | II | 5 | 5+4 | T2N0M0 | Malignant | 1 | 1 | 2 |  |
| PR633 | SHISA2 | F7 | 52 | M | 75 | Prostate | Adenocarcinoma | 3 | III | 5 | 5+5 | T3N1M0 | Malignant | 2 | 1 | 3 |  |
| PR633 | SHISA2 | F8 | 53 | M | 60 | Prostate | Adenocarcinoma | 3 | II | 5 | 5+5 | T2N0M0 | Malignant | 0 | 0 | 0 |  |
| PR633 | SHISA2 | F9 | 54 | M | 60 | Prostate | Adenocarcinoma | 3 | IV | 5 | 5+4 | T3N1M1b | Malignant | 0 | 0 | 0 |  |
| PR633 | SHISA2 | G1 | 55 | M | 63 | Prostate | Adenocarcinoma | 3 | II | 5 | 5+4 | T2N0M0 | Malignant | 6 | 2 | 8 |  |
| PR633 | SHISA2 | G2 | 56 | M | 56 | Prostate | Adenocarcinoma | 3 | II | 5 | 5+5 | T2N0M0 | Malignant | 0 | 0 | 0 |  |
| PR633 | SHISA2 | G3 | 57 | M | 76 | Prostate | Adenocarcinoma | 3 | II | 4 | 4+4 | T2aN0M0 | Malignant | 2 | 1 | 3 |  |
| PR633 | SHISA2 | G4 | 58 | M | 77 | Prostate | Adenocarcinoma | 3 | II | 5 | 5+4 | T2N0M0 | Malignant | 5 | 1 | 6 |  |
| PR633 | SHISA2 | G5 | 59 | M | 75 | Prostate | Adenocarcinoma | 3 | III | 5 | 5+5 | T3N0M0 | Malignant | 4 | 2 | 6 |  |
| PR633 | SHISA2 | G6 | 60 | M | 66 | Prostate | Adenocarcinoma | 3 | II | 5 | 5+5 | T2N0M0 | Malignant | 6 | 2 | 8 |  |
| PR633 | SHISA2 | G7 | 61 | M | 36 | Prostate | Normal prostate tissue | – |  | – | – |  | Normal | 0 | 0 | 0 |  |
| PR633 | SHISA2 | G8 | 62 | M | 37 | Prostate | Normal prostate tissue | – | – | – | – | – | Normal | 0 | 0 | 0 |  |
| PR633 | SHISA2 | G9 | 63 | M | 31 | Prostate | Normal prostate tissue | – | – | – | – | – | Normal | 0 | 0 | 0 |  |
| T195b | TMEM156 | A1 | 1 | M | 64 | Prostate | Adenocarcinoma | 1 | I | 1 | 1+2 | T1N0M0 | Malignant | 7 | 3 | 10 |  |
| T195b | TMEM156 | A2 | 2 | M | 73 | Prostate | Adenocarcinoma | 2 | II | 3 | 3+3 | T2N0M0 | Malignant | 0 | 0 | 0 |  |
| T195b | TMEM156 | A3 | 3 | M | 73 | Prostate | Adenocarcinoma | 2–3 | IV | 4 | 4+4 | T3N0M1 | Malignant | 0 | 0 | 0 |  |
| T195b | TMEM156 | A4 | 4 | M | 61 | Prostate | Adenocarcinoma | 1 | IV | 2 | 3+2 | T3N1M0 | Malignant | 5 | 3 | 8 |  |
| T195b | TMEM156 | A5 | 5 | M | 64 | Prostate | Adenocarcinoma | 1 | I | 1 | 1+2 | T1N0M0 | Malignant | 7 | 3 | 10 |  |
| T195b | TMEM156 | A6 | 6 | M | 73 | Prostate | Adenocarcinoma | 2 | II | 3 | 3+3 | T2N0M0 | Malignant | 4 | 2 | 6 |  |
| T195b | TMEM156 | A7 | 7 | M | 73 | Prostate | Adenocarcinoma | 2–3 | IV | 4 | 4+4 | T3N0M1 | Malignant | 0 | 0 | 0 |  |
| T195b | TMEM156 | A8 | 8 | M | 61 | Prostate | Adenocarcinoma | 1 | IV | 2 | 3+2 | T3N1M0 | Malignant | 5 | 3 | 8 |  |
| T195b | TMEM156 | B1 | 9 | M | 70 | Prostate | Adenocarcinoma | 2 | III | 3 | 3+4 | T3N0M0 | Malignant | 6 | 3 | 9 |  |
| T195b | TMEM156 | B2 | 10 | M | 66 | Prostate | Adenocarcinoma | 2–3 | III | 4 | 3+4 | T3aN0M0 | Malignant | 5 | 3 | 8 |  |
| T195b | TMEM156 | B3 | 11 | M | 65 | Prostate | Adenocarcinoma | 1–2 | II | 2–3 | 2+3 | T2N0M0 | Malignant | 6 | 3 | 9 |  |
| T195b | TMEM156 | B4 | 12 | M | 66 | Prostate | Adenocarcinoma | 3 | II | 5 | 5+5 | T2N0M0 | Malignant | 6 | 3 | 9 |  |
| T195b | TMEM156 | B5 | 13 | M | 70 | Prostate | Adenocarcinoma | 2 | III | 3 | 3+4 | T3N0M0 | Malignant | 3 | 2 | 5 |  |
| T195b | TMEM156 | B6 | 14 | M | 66 | Prostate | Adenocarcinoma | 2–3 | III | 4 | 3+4 | T3aN0M0 | Malignant | 6 | 3 | 9 |  |
| T195b | TMEM156 | B7 | 15 | M | 65 | Prostate | Adenocarcinoma | 1–2 | II | 2–3 | 2+3 | T2N0M0 | Malignant | 6 | 3 | 9 |  |
| T195b | TMEM156 | B8 | 16 | M | 66 | Prostate | Adenocarcinoma | 3 | II | 5 | 5+5 | T2N0M0 | Malignant | 6 | 3 | 9 |  |
| T195b | TMEM156 | C1 | 17 | M | 62 | Prostate | Adenocarcinoma | 3 | II | 5 | 5+4 | T2N0M0 | Malignant | 6 | 2 | 8 |  |
| T195b | TMEM156 | C2 | 18 | M | 69 | Prostate | Low grade malignant leiomyosarcoma | – | Ia | – | – | T1N0M0 G1 | Malignant | 6 | 2 | 8 |  |
| T195b | TMEM156 | C3 | 19 | M | 33 | Prostate | Normal prostate tissue | – | – | – | – | – | Normal | 5 | 3 | 8 |  |
| T195b | TMEM156 | C4 | 20 | M | 43 | Prostate | Normal prostate tissue | – | – | – | – | – | Normal | 3 | 1 | 4 |  |
| T195b | TMEM156 | C5 | 21 | M | 62 | Prostate | Adenocarcinoma | 2 | II | 3 | 3+4 | T2N0M0 | Malignant | 6 | 3 | 9 |  |
| T195b | TMEM156 | C6 | 22 | M | 69 | Prostate | Low grade malignant leiomyosarcoma | – | Ia | – | – | T1N0M0 G1 | Malignant | 5 | 2 | 7 |  |
| T195b | TMEM156 | C7 | 23 | M | 33 | Prostate | Normal prostate tissue | – | – | – | – | – | Normal | 5 | 2 | 7 |  |
| T195b | TMEM156 | C8 | 24 | M | 43 | Prostate | Normal prostate tissue | – | – | – | – | – | Normal | 2 | 1 | 3 |  |
| T195b | TMEM156 | – | – | M | 58 | Skin | Malignant melanoma (tissue marker) | – |  |  |  |  | Malignant | 5 | 2 | 7 |  |

Tissue specimens obtained from US Biomax Inc. (Rockville, MD) for normal (normal tissues from healthy individuals and/or cancer adjacent normal tissues); breast medullary, invasive ductal and lobular carcinomas; liver hepatocellular carcinoma; prostate adenocarcinoma, low grade malignant leiomyosarcoma were subjected to immunohistochemical stain with C11orf68, SHISA2 and TMEM156 antibodies (see methods) (the antibody for G0S2 for immunostaining is not available). The total score is a sum of staining intensity from 0 to 3 (negative to strong) and percentage of cells showing positive staining scored from 0 to 7.

The following tissue arrays from Biomax were used: Breast T088A, BC08013a stained with C11orf68, BC08013 stained with SHISA2, T088a stained with TMEM156; Liver BC03116 stained with SHISA2, T031 stained with TMEM156; Prostate PR633 and T195b stained with C11orf68, PR633 stained with SHISA2 and T1955b stained with TMEM156.
